# Great Expectations: A Critical Review of and Suggestions for the Study of Reward Processing as a Cause and Predictor of Depression

**DOI:** 10.1016/j.biopsych.2020.06.012

**Published:** 2020-06-17

**Authors:** Dylan M. Nielson, Hanna Keren, Georgia O’Callaghan, Sarah M. Jackson, Ioanna Douka, Pablo Vidal-Ribas, Narun Pornpattananangkul, Christopher C. Camp, Lisa S. Gorham, Christine Wei, Stuart Kirwan, Charles Y. Zheng, Argyris Stringaris

**Affiliations:** Section on Clinical and Computational Psychiatry (CompΨ), (DMN, HK, GO, SMJ, ID, CCC, LSG, CW, SK, AS), National Institute of Mental Health, National Institutes of Health; Machine Learning Team (CYZ), Functional Magnetic Resonance Imaging Facility, National Institutes of Health; and Social and Behavioral Science Branch (PV-R), Eunice Kennedy Shriver National Institute of Child Health and Human Development, National Institutes of Health, Bethesda, Maryland; and Department of Psychology (NP), University of Otago, Dunedin, New Zealand.

**Keywords:** Depression, Development, Meta-analysis, Reliability, Reproducibility, Reward processing

## Abstract

Both human and animal studies support the relationship between depression and reward processing abnormalities, giving rise to the expectation that neural signals of these processes may serve as biomarkers or mechanistic treatment targets. Given the great promise of this research line, we scrutinized those findings and the theoretical claims that underlie them. To achieve this, we applied the framework provided by classical work on causality as well as contemporary approaches to prediction. We identified a number of conceptual, practical, and analytical challenges to this line of research and used a preregistered meta-analysis to quantify the longitudinal associations between reward processing abnormalities and depression. We also investigated the impact of measurement error on reported data. We found that reward processing abnormalities do not reach levels that would be useful for clinical prediction, yet the available evidence does not preclude a possible causal role in depression.

Aberrations in how people form expectations about reward and how they respond to receiving rewards are thought to underlie depression. Indeed, there is considerable face validity to the relationship between depression and reward-related processes. Disruptions in reward processing phases such as anticipation, decision, action, and consumption are thought to map onto distinctive depressive symptoms—anticipatory anhedonia (loss of interest in previously pleasant activities), impaired decision making, low energy, and consummatory anhedonia (inability to experience pleasure), respectively (1) (see Figure S1A for an example and detailed description). These reward processes are instantiated in a network encompassing the ventral striatum, the anterior cingulate cortex, and the orbital prefrontal cortex (2). Importantly, work from animal models has shown that lesions in these areas produce anhedonic phenotypes that are characteristic of depression (3,4). Finally, meta-analytic evidence from functional magnetic resonance imaging (fMRI) and electroencephalogram (EEG) studies also shows that altered neural signals in these brain areas acquired during reward tasks are associated with depression (1,5,6). As we review below, the most commonly used task in fMRI studies of reward processing is the monetary incentive delay (MID) task (7), illustrated in Figure S1B. The MID task allows assessment of neural activity related to anticipation or feedback of losses or gains in contrast to neutral trials.

Obviously, the mapping of reward processing onto depressive symptoms could have important implications. It would be clinically useful if tasks that measure reward processing abnormalities (RPAs) could predict onset of depression. Similarly, if RPAs were causally involved, then modifying these abnormalities could help prevent or treat depression. Given this potential, we scrutinized evidence of this relationship and the theoretical claims that underlie it. Therefore, we build on previous reviews that have quantified cross-sectional associations; evaluating the literature in the framework provided by classical work on causality (8) and contemporary approaches to prediction (9). First, we examine the meta-analytic evidence for a cross-sectional association between RPAs and depression. Second, we conduct a new meta-analysis of the longitudinal associations between RPAs and depression. Third, we review evidence on the impact that manipulating reward processing has on depression. Fourth, we consider the challenges of measuring symptoms of depression and reward processing and provide suggestions to address these challenges. Finally, we highlight the conceptual challenges to the purported relationship between RPAs and depression and provide suggestions for improved theoretical framing of future study designs.

## CROSS-SECTIONAL ASSOCIATIONS

RPAs must co-occur with depression in the same individuals if they are to be causes or predictors of the disease. In this section, we critically review meta-analyses of this relationship.

### Functional Magnetic Resonance Imaging

**Figure 1.**
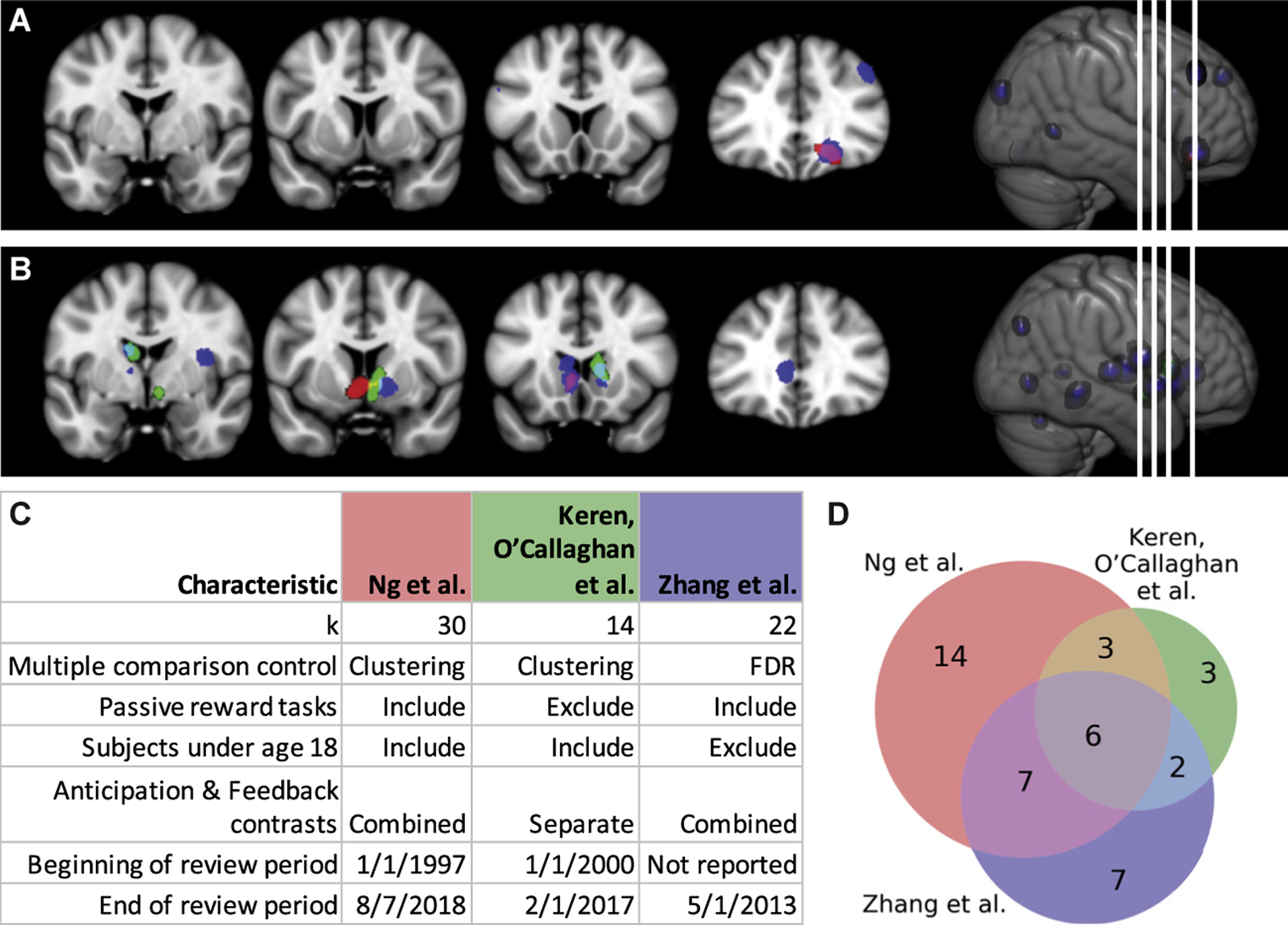
Comparison of coordinate based meta-analyses by Ng *et al*. (5), Keren *et al*. (1), and Zhang *et al*. (6). Results from Ng *et al*. are in red, results from Keren *et al*. are in green, and results from Zhang *et al*. are in blue. Areas of overlap are indicated by additive mixture of the colors in **(A)**, **(B)**, and **(D)**. Results of comparable analyses are consistent in identifying the ventral striatum and caudate as regions that differ during the execution of reward processing tasks between healthy volunteers and participants with major depressive disorder. Keren *et al*. (1) did not find any regions where participants with major depressive disorder showed more activity than healthy volunteers, but both Zhang *et al*. (6) and Ng *et al*. (5) found an area in the orbitofrontal cortex **(A)**. The studies broadly agree on decreased reward responsiveness in the nucleus accumbens and caudate **(B)**. This concordance implies that these findings are relatively robust given the differences in inclusion criteria **(C)** and studies **(D)** included in each meta-analysis. The overlapping regions in **(D)** indicate the number of studies in common between meta-analyses. Of particular note, Zhang *et al*. used a false discovery rate (FDR) for multiple comparison control, which is not as stringent as the clustering approach with a voxel level threshold of *p* < .001 and a cluster-level familywise error rate of .05 used by the other two meta-analyses (105). Keren *et al*. reported results for feedback and anticipation contrasts separately, and here we considered only the results for feedback contrasts. Studies are specified in Table S1.

Coordinate-based meta-analyses test the consistency of the neural location of findings across studies, typically with an approach called activation likelihood estimation (10). Three such meta-analyses have examined cross-sectional differences in reward processing between healthy volunteers and individuals with depression or at high risk of depression (1,5,6). These studies along with their characteristics are presented in Figure 1. While these three analyses included different sets of studies and identified consistent differences in reward-related activity related to depression in a diverse set of regions, they all identified reduced response to reward in the ventral striatum or caudate. Yet, these studies did not provide estimates of the strength of this association. Furthermore, these studies tested only one direction of effect at a time and did not include null effects or effects in the opposite direction, possibly introducing a positive bias.

### Electroencephalogram

In EEG studies, reward processing is assessed by contrasting the signal in response to reward feedback with neutral or loss feedback. Depending on the contrast used, this is called the feedback-related negativity or the reward positivity (RewP). Keren *et al*. (1) meta-analyzed 12 studies that have compared the feedback-related negativity/RewP signal between participants with depression and healthy participants and found a mean effect size (Cohen’s *d*) of .38 (95% confidence interval [CI] = [.12, .64]) across age ranges and a mean effect size of .50 (95% CI = [.15, .85]) in 6 studies on children and adolescents. To give a sense of the potential discriminative capability of this association, we calculated the area under the receiver operating characteristic curve (AUC) [see Box S1 for definition and interpretation of AUC; see Salgado *et al*. (11) for calculation] for these estimates and found that they correspond to an AUC of .61 (95% CI = [.53, .68]) across age ranges and to an AUC of .64 (95% CI = [.54, .72]) in children and adolescents. An AUC of .64 is lower than the performance of even a brief screening questionnaire such as the 2-item Patient Health Questionnaire, which has an AUC of .90 or .88 in younger subjects (12).

In combination, the cross-sectional fMRI and EEG studies to date show that there is a small but consistent difference in reward processing in individuals with depression. The low magnitude of the association means that it would not be a good tool for diagnosing depression but still admits the possibility of a causal relationship.

## LONGITUDINAL ASSOCIATION

**Figure 2.**
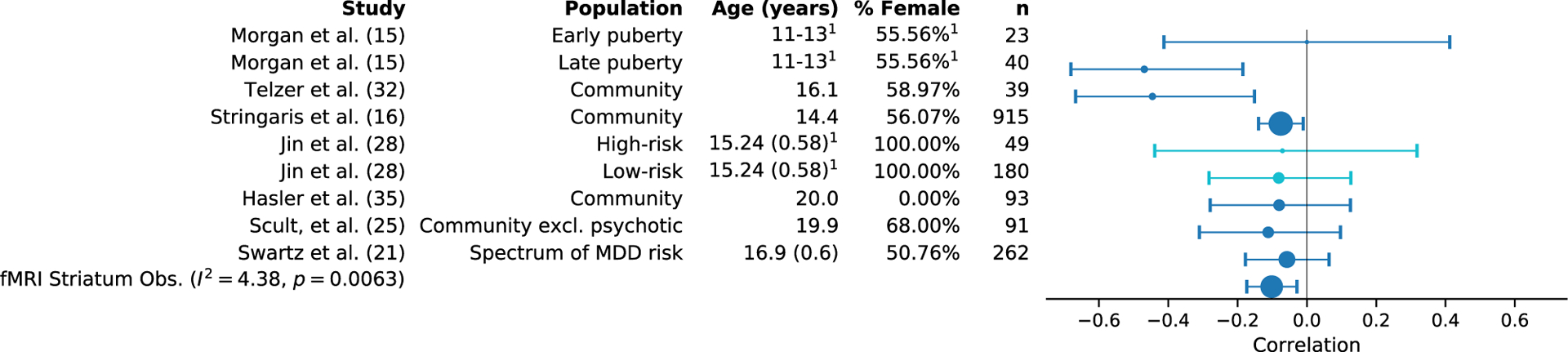
Forest plot for random effects meta-analysis of observational functional magnetic resonance imaging (fMRI) studies reporting a striatal effect for the correlation with change in depressive symptoms. Across these studies (15,16,21,25,28,32,35), predominantly conducted in adolescents, we found that the mean effect size for similar studies was −.10 (95% confidence interval = [−.18, −.03]). In the figure, the size of the marker corresponds to study sample size. The error bars indicate the 95% confidence interval. The results in lighter blue [from Jin *et al*. (28)] represent unreported null effects where the effect size was imputed via the MetaNSUE method. ^1^Demographics reported for the entire study population, not for the subgroup on which the displayed correlation is based. excl., excluding; MDD, major depressive disorder; Obs., observational.

RPAs must precede depression if they are to be a cause of it. Moreover, RPAs could be a prognostic biomarker if they predicted changes in symptoms. Here we conducted a set of preregistered random effects meta-analyses of longitudinal fMRI and EEG studies [(13–35); see also Supplement] to quantify the correlation between neural signals of reward processing and subsequent changes in depression symptoms (see Tables S2–S5 for information extracted from these articles). We imputed nonsignificant unreported effects without introducing bias via iterative maximum likelihood estimation as implemented in the MetaNSUE method (36). We took the strongest striatal or RewP effect from each study, considering observational and treatment studies separately. We found that both striatal fMRI signals (*r* = −.10, 95% CI = [−.18, 2.03], *p* = .0074) (Figure 2) and RewP (*r* = −.18, 95% CI = [−.30, −.04], *p* = .011) are inversely related with changes in depressive symptoms in observational studies (Table 1; see Table S6 for results from treatment studies and Figures S2–S7 for additional forest plots). These estimates are upwardly biased estimates because we used the strongest striatal or RewP effect from each study, combining both region-of-interest and voxel-level results. We also tested a set of global hypotheses in which we took the strongest correlation across the entire brain from each study. We analyzed the absolute value of these correlations because we included activations, connectivity, and psychophysiological interactions. The purpose of these global hypotheses is to define the upper bounds of the relationship between neural reward processing signals and changes in depression symptoms. Based on this, the upper bound for the relationship is .17 (95% CI = [.09, .25]) for observational fMRI studies, with predictions using EEG in a similar range (*r* = .20, 95% CI = [.04, .35]). These associations are large enough to be of mechanistic interest but correspond to AUCs of .60 (95% CI = [.55, .65]) for fMRI and .61 (95% CI = [.50, .70]) for EEG and therefore are unlikely to be useful for prognosis on their own.

There are some limitations to these meta-analyses that may have led to an overestimation of the relationship between RPAs and depression. In particular, only two studies provide out-of-sample tests of prediction accuracy (19,28) (Table S4); the others should be considered tests of within-sample association and would overestimate predictive performance as defined in Box S2. Open science practices improve reliability and reduce positive bias in published reports (37,38), but they are not yet broadly adopted and were not observed in the reviewed studies (Table S5). Finally, we were able to find only a small number of relevant studies and cannot exclude the possibility of study selection bias.

### Implications of Measurement Error for Estimating the Relationship Between Reward Processing and Depression

**Figure 3.**
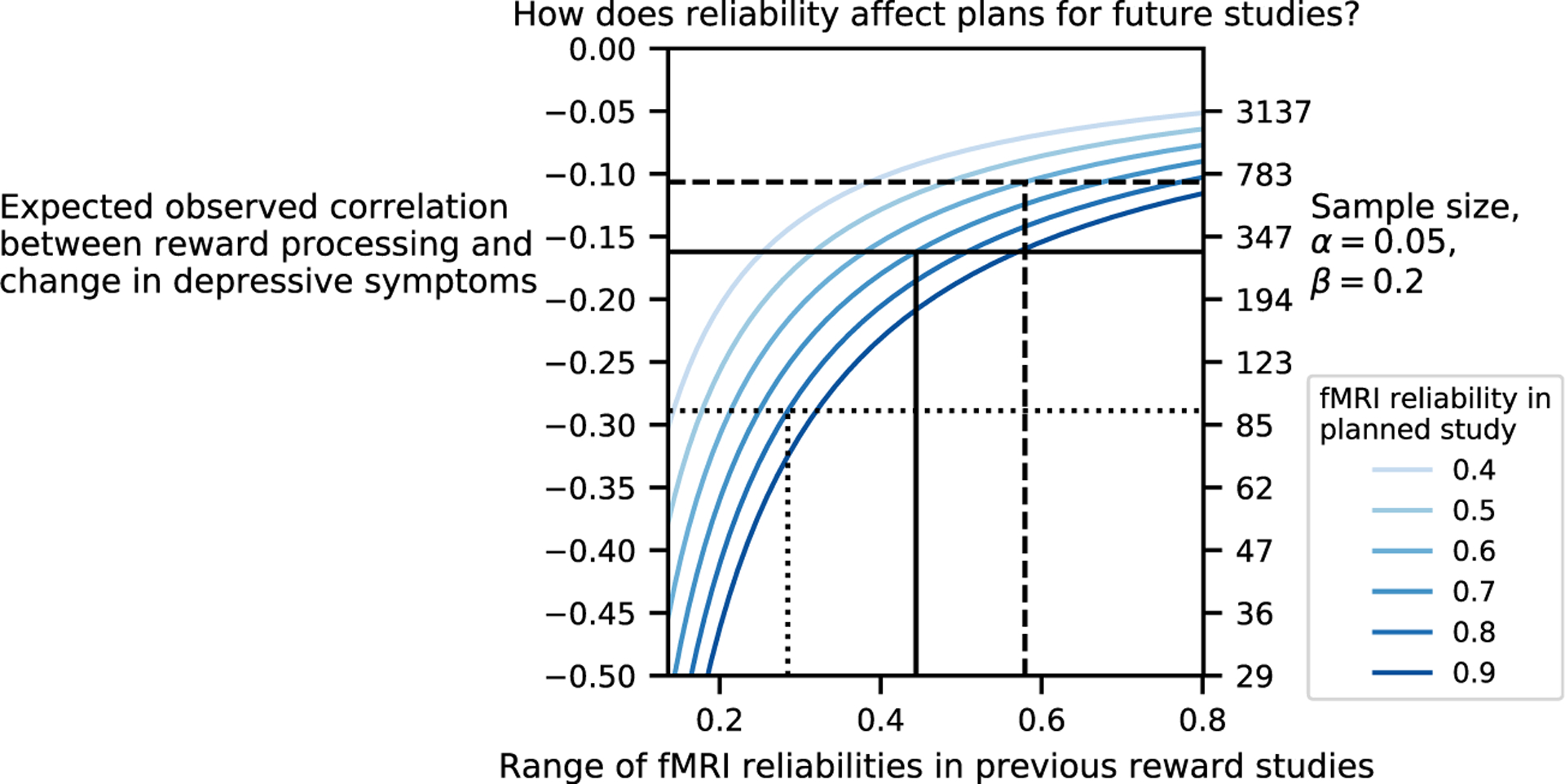
Impact of functional magnetic resonance imaging (fMRI) test–retest reliability on expected effect size and required sample size of future studies. The figure depicts the dependence of the effect size to be expected in future studies (y-axis on the left) and the sample size of future studies (y-axis on the right) on the reliability of past studies (x-axis) and the expected reliability of future studies (color-coded lines). The reliability of past studies is derived from references (39–45), and the minimum and maximum values bound the x-axis. The following 3 cases are illustrated. Dotted line: If previous studies had a low reliability (.28) and our planned study will have a reliability of .80, we would expect to observe a correlation between reward processing and change in depressive symptoms of −.29 (95% confidence interval [CI] = [−.52, −.02]) and would expect a required sample size of 92 to have 80% power to detect with a two-sided test for Pearson correlation difference from 0. Solid line: If previous studies had a reliability of .44 and our planned study will increase this to .70, we would expect an observed correlation of −.16 (95% CI = [−.31, −.01]), requiring a sample size of 297. Dashed line: If previous studies had a reliability of .57 and our planned study will have a reliability of .60, we would expect an observed correlation of −.11 (95% CI = [−.21, −.01]), requiring a sample size of 690. For this figure, we assume a depressive symptom measurement reliability of .77 (95% CI = [.67, .84]) based on 8 studies (Table S8). If depressive symptom measurement reliability is improved in the planned study, the above observed effect sizes would be proportionally higher (Figure S8).

Here we demonstrate that our current ability to estimate the correlation between RPAs and change in depression is affected by measurement error, operationalized as the reliability of a measure. Importantly, measurement error has profound implications for sample size required for future studies. To assess the degree of measurement error in studies reviewed in the previous section, we estimated the reliability of past fMRI studies of reward in a random-effects meta-analysis of test–retest reliability across the 9 reward-related fMRI analyses (39–45) from Elliott *et al*. (39) (median *n* = 25, interquartile range = 5; median test–retest interval = 14 days, interquartile range = 20 days) (Table S7). We found the test–retest reliability to be .44 (95% CI = [.28, .58]). Using these estimates, we conducted a power analysis for future studies as a function of future fMRI reward signal reliability. We compute the expected future observed effect size in two steps. First, previous effect sizes need to be reliability corrected by dividing the effect size by the reliability of the measures used in these studies. Then, to obtain the expected observed effect size in a future study, the reliability-corrected effect size needs to be multiplied by the anticipated reliability (see Supplemental Methods for more details). Figure 3 demonstrates how expected effect sizes and, as a result, required sample sizes are affected by past reliability (x-axis) as well as assumed future reliabilities. The figure also makes clear why investing in improving fMRI reliability in future studies is crucial (see shifts in required sample sizes given different future reliabilities).

Our meta-analysis focused on univariate analyses to facilitate comparisons across studies, but multivariate methods are a promising approach to improve reliability (46,47). These methods may pool information across multiple regions, multiple phases of reward processing (14,31), multiple modalities, or all of the above (34). There are many approaches in machine learning to combine multiple predictors (48), including regularized regression, random forests, and deep learning, but application to prediction of future disease severity remains rare. The analogy to similar approaches in genetics may help explain this; just as single nucleotide polymorphisms have proven to have small individual effects, but polygenic risk scores have proven to be useful for prediction (49), so could neural predictors of disease severity benefit from multivariate approaches.

In sum, there is evidence that reward processing signals correlate with changes in depression symptoms. This correlation is consistent with the hypothesized mechanism of RPAs causing anhedonia and depression.

## MANIPULABILITY

If RPAs cause depression, then altering the reward processing network should alter the clinical phenotype and course of depression. Manipulating reward stimuli changes ventral striatum activity as well as subjective ratings of momentary mood (50,51). However, evidence that manipulating the reward processing system changes clinical symptoms of depression has been largely elusive. The ideal evidence would come from a randomized, placebo-controlled trial where the intervention is shown to cause a change in reward processing and, consequently, a change in behavior. Statistically, this amounts to a mediation. Most studies that use pharmacological manipulations have so far demonstrated primarily that interventions perturb the reward system. Such interventions are typically designed as acute-dose trials of a drug and have included serotoninergic (52,53), dopaminergic (31,54–59), cannabinergic (60,61), glutamatergic, and opioidergic (62) manipulations. There is also preliminary evidence from small, open-label trials that deep brain stimulation of the nucleus accumbens treats depressive symptoms (63–66) and normalizes nucleus accumbens responses to reward (66). Several treatment trials have shown that changes in the reward system correlate with changes in depressive symptoms. In a placebo-controlled, double-blind crossover trial in 36 patients with treatment-resistant depression, Lally *et al*. (67) demonstrated that ketamine specifically lowered anhedonia and showed that increases in striatal glucose use correlated with changes in anhedonia. EEG markers of reward processing have also been shown to correlate with changes in depression and anxiety symptoms during treatment with cognitive behavioral therapy (*n* = 34) and selective serotonin reuptake inhibitors (*n* = 29) (29). Other small studies have found correlations with treatment response to pharmacological interventions (*n* = 15) (68) and psychotherapy (*n* = 15 in each study) (69,70). On the other hand, there is at least one example of a pharmacological intervention (a κ-opioid antagonist) that increased striatal response to reward but did not change symptoms relative to placebo (71). Only one study has reported mediation; a double-blind trial of sertraline (which targets both dopaminergic and serotonergic systems) in 222 adults found that an fMRI-derived index of striatal reward processing mediated the effect of sertraline on depressive symptoms (14). This provides some evidence for the manipulability of depression symptoms via manipulations of the reward processing system, supporting the possibility of a causal relationship.

## MEASUREMENT CHALLENGES

### Measurement of Reward Processing

Several experimental approaches have been developed to isolate components such as anticipation and consummation of reward. Many behavioral tasks correlate poorly with self-report measures owing to low reliability and measurement of different entities (72). In addition, some widely used neuroimaging tasks, such as the MID (7,73), lack a behavioral output. Interpreting blood oxygen level–dependent (BOLD) signal in the absence of behavior is fraught with ambiguities; a reduced BOLD signal could be a deficit or a compensatory mechanism.

Most studies employ tasks that measure only some of the components of reward processing. For example, in the MID, the most commonly used task, only prediction (measured as neural activity during the anticipatory period) and experience (measured as neural activity during the feedback period) of reward are probed (Figure 1), while other important phases such as decision and effort are left out (74). This means that key components of the reward system are not probed in the same individuals, and therefore inferences drawn about reward processing may be biased or partial. Computational modeling [as in (50,75)] of all the phases of reward, potentially across multiple tasks within the same individuals, would allow a more thorough phenotyping of the reward system (76,77).

### Multiplicity of Measurement

Different neuroimaging studies define the same phase of reward processing in different ways. For example, the label reward anticipation is applied to analyses that contrast it with a neutral condition, a loss condition, or even just baseline activity. In the fMRI studies reviewed in the meta-analyses described above (1,5,6), we found 19 different tasks, 14 of which have been used no more than twice (Figure S9 and Table S1). Across these tasks, at least 69 different task–contrast combinations were used, 54 of them only once. The most commonly reported was the gain anticipation versus neutral anticipation contrast for the MID task in 10 studies. Given such a large space of potential tasks, contrasts, and analytical approaches, it is impossible to know whether the contrasts and analyses used in any given article are the only analyses done or whether they are the result of searching that space for a significant finding (78,79). This may lead meta-analyses like those above to overestimate the magnitude of the relationship between reward processing and depression.

### Measurement of the Clinical Phenotype

Diagnosis of major depressive disorder is based on self-report of subjective symptoms (80), which presents several challenges as previously discussed (81,82). In particular, there are inherent problems with self-reported anhedonia, in particular consummatory anhedonia, or the lack of enjoyment when experiencing a reward. In our introductory example (Figure S1A), the child, sitting in a research laboratory, would be asked about her experience of consuming the chocolate. This requires forming the mental representation of a past event and attaching value to it, a different process than that of actual consummation and in some ways more related to the process of predicting the value of a future reward based on past experiences rather than reporting on the actual experience. This is especially problematic because patients with depression may have different recall biases compared with healthy volunteers (83). Ecological momentary assessment may allow more direct measurement of consummatory anhedonia (17,18), and assessment of effort expenditure or neural responses to reward delivered in the scanner may be another way to characterize consummatory anhedonia (84,85).

### Proposed Solutions

Measurement of reward processing, depression, and anhedonia is challenging, but these are obstacles we must overcome as a field if we are to understand the relationships among these constructs. We propose a broad collaborative effort unifying a behaviorally informative task or tasks, measures of clinical symptoms, and generative computational models to address these challenges as outlined in Table 2. This is certainly not an exhaustive inventory of the goals such an effort would need to achieve, but we hope that it will serve as a starting point for the creation of a more robust set of tools for understanding reward processing, depression, and their relationship.

## CONCEPTUAL CHALLENGES

For a promising and well-studied topic such as RPA and depression, there are surprising gaps in our theoretical framework about its origins, directions of effect, and specificity. We discuss these below along with proposals for solutions.

### Origins of RPAs and Depression

So far, we have focused on the possibility of a causal relationship between RPAs and depression, but we have not considered the origins of RPAs themselves. The associations between depression and reward processing described above could be due to genes, environment, or their interplay. Small preliminary twin studies have suggested that striatal responses to reward and risk are moderately heritable (86,87). Animal and human studies have demonstrated that stress can reduce striatal reward responses (88–93). The relationship may be more complicated in that a genetic predisposition to RPAs may represent a vulnerability to depression when exposed to a stressful environment (23,30,94). An immediate research need is a twin study to test the genetic and environmental origins of the covariation between depression and reward processing.

Development is another important factor to consider in the origins of RPAs and depression. A dramatic rise in new depression cases occurs during adolescence (95), coinciding with a period of time when, normatively, adolescents are apparently more sensitive to rewards (96). Moreover, there is some meta-analytic evidence (1) to suggest that RPAs in depression may be more pronounced in adolescents compared with adults. Yet, with notable exceptions (15,97,98), rarely are specific theories being proposed about the interplay of development with reward processing and depression. It is even rarer to see any robust tests of such theories (15). For example, one possibility is that neural reward responsiveness may be an important depression-related diathesis that interacts with other genetic and environmental factors at sensitive developmental stages such as puberty.

### Direction of Relationship

**Figure 4.**
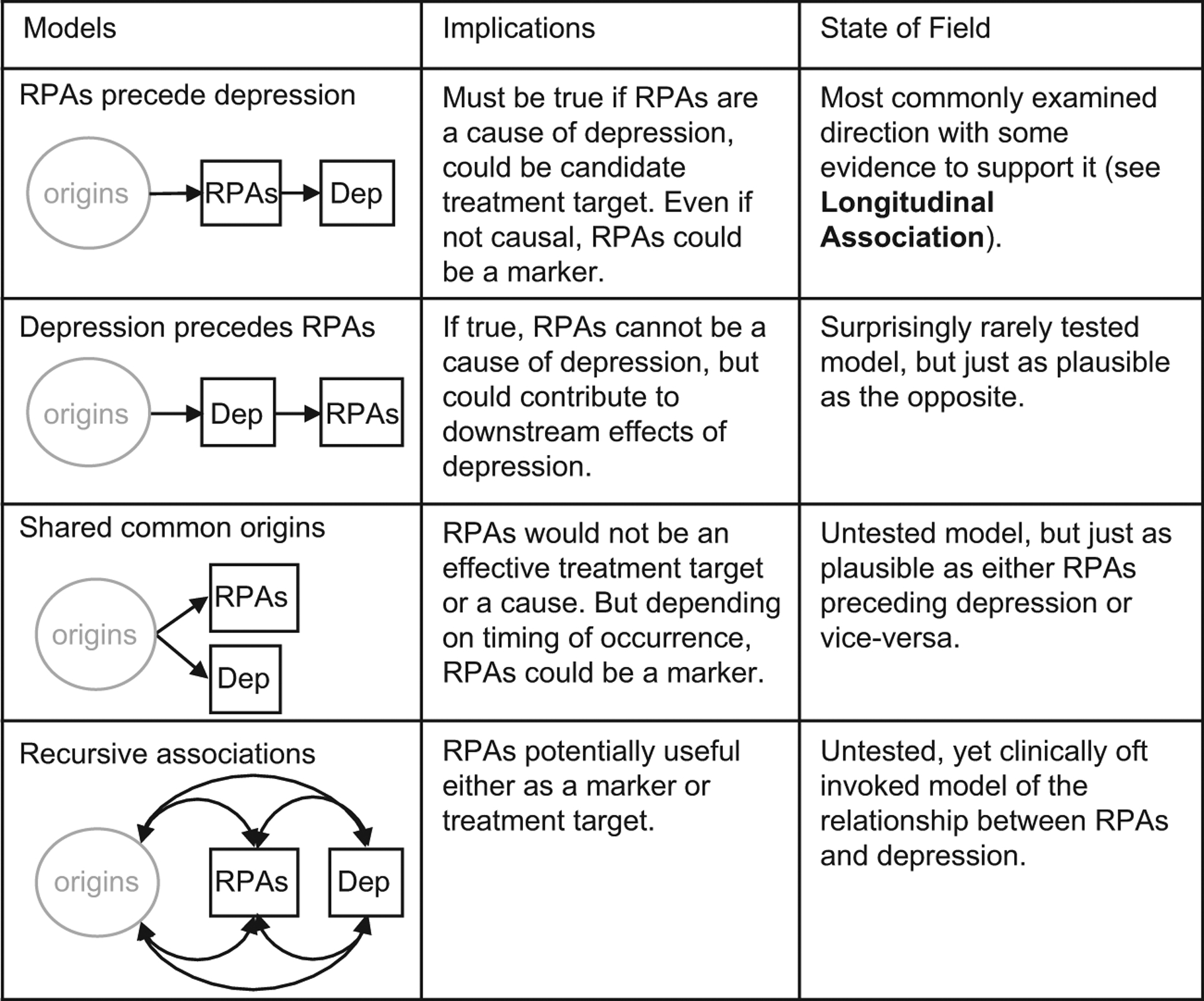
Schematic depiction of potential relationships between depression (Dep) and reward processing abnormalities (RPAs). The most commonly tested model is that RPAs precede depression, but plausible alternatives such as the reverse of this relationship or a common origin receive little attention. The recursive associations model is frequently cited in the behavioral analysis literature (106–108) but has not been thoroughly tested. Datasets from many of the studies cited in the “Longitudinal Association” section could be used to assess the relative strengths of RPAs preceding depression versus depression preceding RPAs. In the schematics, “origins” indicates an unspecified combination of genetic and environmental factors. This is by no means a depiction of every possible model; mediating and moderating relationships, for example, are not depicted.

The direction of the relationship between RPAs and depression is critical, yet studies rarely assess it (Figure 4). The majority of longitudinal studies of the relationship between reward processing and depression have examined the hypothesis that RPAs precede depression, but other plausible models are not as commonly considered. Depression could precede RPAs and the causal relationship could in fact be the reverse. Indeed, it could be that depression affects reward anticipation or enjoyment and that this is responsible for some of the downstream effects of depression such as social isolation. Another possibility is the existence of a shared risk factor causing both depression and RPAs. In such a case, the relationship between the two would be of little consequence for intervention or prediction. Finally, clinicians, particularly in the behavioral activation literature, often invoke a recursive relationship between RPAs and depression. Ideally, these explanatory models (Figure 4) should be tested against each other (see Box S2). However, we are not aware of data collected with sufficient temporal density and applying the necessary modeling to demonstrate the superiority of any of these models.

An alternative focus is prediction, for example, testing whether baseline neural reward signals improve the prediction of future depression beyond what other inexpensive and commonly available symptom measures and demographic factors contribute, termed incremental validity (Box S2). In this case, it might not matter whether there is a common latent cause of RPAs and depression; RPAs could still be a predictor of depression if they respond more quickly to changes in that latent factor. In evaluating both explanatory models and predictions, stronger evidence is provided by preregistered hypotheses and analytical plans (99).

### Specificity

If RPAs are not specifically related to depression, they are less likely to be a specific cause of depression or a specific clinical predictor. There are several alternative hypotheses that have only partially been tested. First, within depression there is some evidence that RPAs are specifically related to anhedonia. Three studies have found that anhedonia, but not low mood, is related to RPAs (16,84,100). Yet comparing anhedonia with other plausible symptoms, such as loss of energy and fatigue, has yet to be done. Moreover, studies do not take comorbidity between depression and other disorders into account. It is possible that within depression the distribution of symptoms from other disorders (e.g., social anxiety symptoms, particularly in adolescents) moderates the association between depression and reward processing (96).

Second, anhedonia (and reduced striatal BOLD signal during reward anticipation) is present in other common mental disorders, including schizophrenia and attention-deficit/hyperactivity disorder (ADHD) (101,102). In some studies of schizophrenia, this signal has been accounted for by depression comorbidity (103); in ADHD, this reduction was observed only in adult samples but not in youth samples (43). In a recent study from our group, reduction in striatal activity was observed only in children with anhedonia but not in those with anxiety or ADHD in a community sample (while ADHD was associated with BOLD signal aberrations during a working memory task) (100). Anhedonia has been suggested as a transdiagnostic symptom (74). This may be true but has not been tested yet in a way that would satisfy criteria for trans-diagnostic research (104). Future studies linking anhedonia with RPAs across patient populations are needed to understand the specificity of this relationship.

### Proposed Solutions

We do not currently have the kind of published studies that would be needed to address these conceptual challenges. Differentiating the role of genetics and environment on reward processing, finding the most likely direction of effect between reward processing and depression, and characterizing the specificity of RPAs to depression all require, as a base, a densely sampled longitudinal study (Table 2). A longitudinal design where reward processing and depression are characterized at least four times would provide several benefits. It potentially would allow us to distinguish between potential directions of effect, estimate the reliability of clinical and imaging measures within the same study, improve precision of measures through multiple measurements, and estimate nonlinear (e.g., quadratic) trajectories of imaging or clinical signals. Adding twin and family studies and characterization of stressful life events to this design would allow us to address questions about the relative contributions of genetics and environment to the development of RPAs and depression. Conducting a densely sampled longitudinal study in a trans-diagnostic population would allow us to address questions of the specificity of RPAs to depression.

### Conclusions

Neural RPAs are currently unsuited for use as clinical predictors of depression, but improved measures of neural signals of reward processing and multivariate analyses may change this in the near future. There is evidence to support a causal relationship between RPAs and depression, with weak temporal association and evidence for manipulability. We have made general suggestions for improving the measurement of reward processing and depression, and we have proposed experimental designs (Table 2) for addressing some of the conceptual challenges we observed in the literature. Not all these suggestions are applicable to every study of reward processing and depression, but we hope that they will be a useful guide to the design of future studies.

## Supplementary Material

1

## Figures and Tables

**Table 1.** Summary of Longitudinal Meta-analytic Hypotheses

Modality	Specificity	Design	*k*	*r* [95% CI]	*z*	*P*	*I* ^2^	Worst *r*	Worst *z*	Worst *p*
fMRI	Striatum	Obs.	9	−.10 [−.18, −.03]	−2.64	.0074	4.57%	−.08	−2.24	.025
EEG	RewP	Obs.	5	−.18 [−.30, −.04]	−2.63	.011	74.41%	−.11	−2.07	.038
fMRI	Global	Obs.	13	.17 [.09, .25]	4.30		51.45%	.15	3.76	
EEG	Global	Obs.	5	.20 [.04, .35]	2.54		81.46%	.12	2.19	

The global results are best-case analyses taking the absolute value of the strongest effect from any reward-related analysis to define the upper bounds of the relationship between reward processing and future changes in depression. No *p* values are given for global results because significant difference from 0 is trivial after taking the absolute value. The results shown here are from observational studies; results from treatment studies are shown in Table S6. The least significant results from a leave-one-out analysis are shown in the “worst” columns. The results of the leave-one-out analysis indicate that these correlations would be reduced, but still likely different from 0, if the most significant study were removed from the analysis in each case.

CI, confidence interval; EEG, electroencephalogram; fMRI, functional magnetic resonance imaging; Obs., observational; RewP, reward positivity.

**Table 2.** Measurement and Conceptual Challenges

Challenge	Suggestion
Measurement Challenges	
Uninformative behavioral outputs	The behavioral outputs of the task should be sensitive to intraindividual change with good test–retest reliability (72).
Measuring only some phases of reward processing	The task should assess all or many phases of reward processing in tandem.
Measuring only some clinical features of anhedonia	The assessments should measure multiple aspects of anhedonia, ideally in a nonretrospective or vicarious way, to disentangle recall of reward from actual anticipation or experience of reward. This may entail both questionnaires and ecological momentary assessment.
Not clearly linking task outputs and neural correlates with specific symptoms	Computational models of the task should explicitly represent the theorized relationships between the phases of reward processing and symptoms/types of anhedonia so that these relationships can be tested.
Many possible analytical choices	The task should have a core set of contrasts that are reported in every study used to facilitate future metaanalyses; of course, additional contrasts and analyses would be welcome.
Many possible tasks and questionnaires	There are several steps that should be taken to promote widespread use so that the creation of another task and questionnaire does not simply exacerbate the already fractured landscape. The task 1) should be developed collaboratively [similar to the model used in the development of the Brain Imaging Data Structure (109)] to promote use and adoption; 2) should be amenable to repeated administrations in longitudinal studies; and 3) should be accessible to developmental samples.
Conceptual Challenges	
Origins of reward processing abnormalities and depression	A densely sampled longitudinal design with twins and/or families should be used. Stressful life events and other aspects of the environment should be measured.
Direction of effects	A densely sampled longitudinal design would serve as the basic framework in which to differentiate the possible directions of effect. Employing this design in the context of an intervention would test the possibility of a latent factor that influences both depression and reward processing.
Specificity	A densely sampled longitudinal design in a cross-diagnostic population would allow testing of the hypothesis that reward processing is a transdiagnostic feature of psychopathology.

Under Measurement Challenges, we propose a collaborative effort to develop a reward processing task (or battery of tasks), anhedonic symptom assessments, and generative computational model in concert and describe how it will meet the challenges of measuring reward processing and anhedonia. Under Conceptual Challenges, we propose a densely sampled longitudinal design and modifications to this basic design required to answer the conceptual challenges to understanding the relationship between reward processing abnormalities and depression.
