# Supplementary material for: Great Expectations: A Critical Review of and Suggestions for the Study of Reward Processing as a Cause and Predictor of Depression
Source: Biol Psychiatry. Author manuscript; Available in PMC 2023 Dec 18. (PMC10726343; doi:10.1016/j.biopsych.2020.06.012)
Supplement: 1 [file NIHMS1604864-supplement-1.pdf]

Great Expectations: A Critical Review of and Suggestions for the Study of Reward Processing as a Cause and Predictor of Depression

Supplemental Information

Supplemental Methods

Preregistrations and deviations

We preregistered the approach to our literature review (https://osf.io/be4nt, https://osf.io/mp49y) and meta-analysis (https://osf.io/3dz54). The first registration (https://osf.io/be4nt) described our methodology, repositories, search terms, and inclusion/exclusion criteria. The second was a note modifying the search period from going through the end of 2019 to stopping at December, 17th, 2019 due to the limited search granularity provided by many of the publication databases we used. We added a second round of title and abstract screening in addition to the single round specified in these registrations and had two separate investigators (AS and DMN) review articles for inclusion or exclusion.
The last registration (https://osf.io/3dz54) is for the analytical and qualitative approach for the meta-analyses. This registration was completed after reviewing papers for inclusion, but prior to beginning data extraction for the meta-analysis. In our registration we referred to feedback negativity, which is the subtraction of gain ERP from loss ERP. In reviewing the studies, we found that reward positivity (RewP, the subtraction of loss ERP from gain ERP) was more commonly reported and so we use RewP here. In our registration we stated that we would only use MetaNSUE if non-significant unreported effects made up 20% or more of the relevant effects to a particular hypothesis, but we instead used it for all analyses for consistency.

Meta-analysis

Literature review: We searched PubMed, Scopus, PsycINFO, and Web of Science for articles published in English from February 1, 2017 to December 17, 2019, using the following terms and their derivatives: depression, anhedonia, reward, motivation, reinforcement, punishment and aversion, prediction error, decision making, and risk taking. This is the specific query:
(((((depress*) OR (anhedon*)) AND ((reward*) OR (motiv*) OR (reinforc*) OR (punish*) OR (aversi*) OR ("prediction error") OR ("decision making") OR ("risk taking")))) AND ("2017/02/01"[Date - Publication]: "2019/12/31"[Date - Publication])) AND English[Language]
Studies returned by this initial query were reduced by removing duplicates and non-human and non-experimental studies based on keyword searches. 30 articles were randomly selected from the remainder and screened for inclusion/exclusion by independent investigators (DMN, CCC, SK, CW, SMJ, LG). The inter-rater reliability was found to be 0.88 and the remaining sample of studies was divided among the investigators for abstract screening. Each article was screened twice and those that passed either screening were divided up among the investigators for a more thorough review by two investigators (AS and DMN). Our literature review covered the period starting February 1, 2017 because it is an extension of the literature review performed in Keren, O'Callaghan *et al.* (1), which reviewed publications from January 1, 2000 to February 1, 2017, thus the two combined reviews cover January 1, 2000 to December 17, 2019. We identified 13 potential longitudinal papers from Keren, O'Callaghan *et al.* that were also given a thorough review. These two investigators decided together which articles to include and exclude.

Inclusion criteria: To be included, studies had to provide a measure of depression or anhedonia in people with major depressive disorder, in people at high risk of depression, or in healthy volunteers. We selected only studies that measured depression or depressive symptoms through questionnaires, structured interviews, or clinical diagnosis. In terms of reward paradigms employed, and following the classification described by Richards et al. (2), we included instrumental- reward tasks and decision-making tasks, which require participants to complete an action correctly in order to obtain a reward, as this action is linked to the reward value at a trial- by-trial level. Hence, reward paradigms in which rewards were presented passively were excluded. Either positive (e.g., winning money) or negative (e.g., losing money) reward manipulations were permitted. No age restrictions were applied. In addition, studies must have reported analyses examining the ability of measures collected at one time point to predict severity (as evidenced by changes in depression/anhedonia status or scores) or treatment response at a subsequent time point.

Exclusion criteria: Studies were excluded if they lacked a standard measure of depression. We excluded studies that measured depressive symptoms in patients with a different primary disorder (e.g. bipolar or schizophrenia, etc.), but did not include in addition a depressed group. This was done because our primary question concerns the effects of depression on reward processing and in the absence of a depressed control group, drawing inferences about such effects would be impossible. We did not exclude studies in which patients with depression also had co-morbid anxiety disorders. We also excluded studies in which reward processing was only measured through non-experimental methods such as self-report measures or questionnaires. We excluded studies in which physical punishment was delivered (e.g. heat, pain, electrical shock, etc.) as these are likely to engage different brain networks. This would have included any studies in which physical pain and reward are tested in separate tasks and analyzed separately, but we are not aware of any studies excluded this way. For included studies, relevant methodological details, where available, were recorded, as outlined below.

Data Extraction: We only evaluated one prediction (*i.e.* longitudinal association) for each set of study participants. If multiple predictions were reported for the same set of subjects we picked the prediction over the longest time period, if multiple predictions were made with the same time period, we picked the highest quality prediction which used the most participants. Each study could potentially provide more than one prediction if they reported separately on different samples.
We rated the quality of each longitudinal prediction based on the criteria put forward in Poldrack et al. (3). Specifically:

- Reporting of out of sample model fit indices
    - Least risk for a completely separate evaluation set. Highest risk for in-sample model fit indices reported as predictive performance.
        - 0: completely separate test set
        - 1: within sample cross validation
        - 2: in-sample model fit reported as predictive performance
- Cross-validation procedure that encompasses all analytical manipulations
    - Highest marks if all normalizing and selection done separately for each validation fold. Lowest if only the final model fit is done in a cross validated fashion.
        - 0: all cross-dataset analytical procedures performed within training set
        - 1: some analytical procedure performed within the training set, but not all
        - 2: significant chance of data leakage between training and test

- Sample size
  - Ideally greater than several hundred observations
    - 0: > 200
    - 1: > 50 < 200
    - 2: <50
- Reporting multiple measures of model fit
  - Lowest risk if multiple measures such as R^2 in addition to measures of unsigned error like mean square error or mean absolute error are provided. Highest risk if none of these measures is provided.
    - 0: provided at least 2 measures of predictive performance, one of which is MSE or absolute error
    - 1: only r-squared or a single metric reported
    - 2: no standardized predictive accuracy reported
- Calculating coefficient of determination via sum-of-squares formulation
  - Lowest risk for sum-of-squares formulation. Highest risk for squared correlation coefficient.
    - 0: sum-of-squares formulation
    - 2: squared correlation coefficient
- Use of k-fold or shuffle-split cross validation
  - Lowest risk for shuffle-split or k-fold cross validation with k between 5 and 10. Highest risk for leave-one-out cross-validation.
    - 0: K-fold or shuffle split cross validation with k between 5 and 10
    - 1: some intermediate cross validation scheme
    - 2: leave one out cross validation

In addition, we evaluated the open science practices of the publication on the following criteria:

- Use of pre-registration
  - 0: if a link to a thorough pre-registration is provided
  - 1: if link is provided but there are extensive deviations from the preregistration
  - 2: no pre-registration
- Publicly releasing code or scripts used in analysis
  - 0: link to working code provided
  - 1: link provided, but code does not work
  - 2: no link to code
- Publicly releasing data used in analysis
  - 0: raw data available with or without a data-use-agreement
  - 1: statistical maps or other derivatives released
  - 2: no data released

Each criteria was rated on a scale of 0 for least risk of bias to 2 for high risk of bias as described above, or N/A, for example the K-fold criteria is not applicable if no cross-validation was performed. Quality was evaluated by two separate raters (DMN and GO’C), conflicts were reconciled in person and are reported in Table S4.

Two raters (DMN and GO’C) independently extracted the information described below from each longitudinal prediction.

- Observational or treatment
- Study in which source data were first described

- Nature of each group (healthy, at risk (defined as the presence of either MDD in a parent, high depression scale scores in the absence of MDD diagnosis, or remitted MDD, depressed), participants with another disorder)
- Criteria used for diagnosis if relevant
- Sample size of each group
- Percentage of females in each group
- Percentage of medicated individuals in each group
- Mean, SD, and range of ages in each group
- Neural measure: EEG, functional connectivity, fMRI
- Depression measure
- Reward task
- Type of reward (monetary, affective, or primary)
- Contrast used (if any)
- Prediction interval in time
- Terms used in the predictive model
- Link to preregistration
- Link to code
- Link to data
- Treatment type (pharmacological or psychological)
- Specific treatment

For fMRI studies we separately extracted information for the location/connection providing the best predictive performance across the entire brain and the striatal location/connection with the best predictive performance:

- Direction of effect
- Reported statistic
- Predictive effect size uniquely contributed by the neural information
- Overall predictive effect size
- Template space of reported components
- Coordinates of effect
- Connections

For EEG studies extracted information for both the most predictive component of the EEG signal and for the RewP:

- Electrodes sampled from
- Type of signal extracted (FRN, RewP, etc.)
- Sampling method (mean amplitude, peak, etc.)
- Window for sampling
- High and low pass filter applied
- Reference electrode(s)
- Type of EEG cap or net used
- Direction of effect
- Reported statistic
- Predictive effect size uniquely contributed by the neural information
- Overall predictive effect size

Reported effects were transformed to have a uniform direction of effect as appropriate, for example, the sign of feedback negativity results were flipped so that they were in terms of RewP. Reported effects were transformed into correlation coefficients reflecting the unique contribution of neural data. Transformations from t or F statistics to correlation coefficients based on the following formulas (4):

$$r = \sqrt{\frac{t^2}{(t^2 + df)}}$$

$$r = \sqrt{\frac{df_n F}{df_n F + df_d}}$$

df: degrees of freedom; df_n: numerator degrees of freedom; df_d: denominator degrees of freedom
Degrees of freedom were adjusted appropriately for the number of regressors. When only a beta coefficient was reported, this was converted to a t by dividing by the standard error and then from t to correlation coefficient.

Analytic approach: We sought to test the central hypothesis that the expected effect size for a study predicting future depression severity from neural reward-related signals is not 0. We operationalized this hypothesis into 8 separate hypotheses based on the modality (fMRI or EEG), specificity (striatal reward contrast/RewP or any signal), and study design (treatment or observational). We established an a priori minimum sample size of 5 predictions for each of these hypotheses. We performed random-effects meta-analysis of correlation coefficients using Fisher's transform with multiple imputation of non-significant unreported effects as implemented in the R package MetaNSUE (5,6). We rely on the tables converting effects sizes to area under the receiver operator characteristics curve (AUC) provided by Salgado (7) to help put the effect sizes we report in a form more familiar to machine learning practitioners. Correlation coefficients are first converted to d before converting to AUC.

Accounting for the effect of measurement error on observed correlations

After completing our meta-analysis, we wanted to understand how our findings constrained the distribution of reliability-corrected values for the correlation between neural reward processing signals and future depressive symptoms given that both depressive symptoms and reward processing are subject to measurement error. We first estimated the test-retest reliability of neural reward signals and depressive symptoms, then we used the algebraic relationships derived below to calculate the reliability relationship between neural reward processing signals and future depressive symptoms based on the meta-analytic estimate of correlation between striatal fMRI reward signal and change in depression symptoms in observational studies. Finally, we reversed the calculation to determine how measurement error in future studies would impact the expected observed effect size.

Estimating measurement error of neural signals: We extract the intra class correlations, sample sizes, and measurement intervals from Elliott et al. (8) (Supplemental Table 7) for reward-related tasks with a prediction interval of less than 100 days. This resulted in 9 values from 7 studies from which to meta-analytically estimate the reliability of neuroimaging measures of reward. We then ran a random effects meta-analysis to determine the mean correlation, standard error of that estimate, and an estimate of the between studies heterogeneity, τ^2.

Estimating measurement error of depression symptoms: We conducted an informal literature review to identify studies that assessed test-retest reliability of the measures of depression used in the studies in our longitudinal meta-analysis. We extracted the correlation coefficient, sample size, prediction interval, and description of the population from studies that examined test-retest reliability of depression measures over an interval of less than 100 days (Supplemental Table 8). This gave us 13 reliability estimates for 6 measures from 6 studies. We then ran a random effects meta-analysis to determine the mean correlation, standard error of that estimate, and an estimate of the between studies heterogeneity, τ^2.

Deriving relationship between measured and reliability-corrected correlation: We algebraically derived the relationship between between the above test-retest reliabilities (neural signals of reward processing: r_{NNm}; depressive symptoms: r_{DDm}), the measured relationship between neural reward signals and change in depression symptoms (r_{NmDm}), and the reliability co relationship between neural reward signals and change in depression symptoms (r_{ND}). In order to do so, we assume an additive model for noise, such that the covariance between the underlying constructs and their measurement is 1 and the standard deviation of the underlying construct is 1. This simplifies the relationship between test retest correlation and the standard deviation of the measured properties. Formally:

$$r_{NN_m} = \frac{cov(N, N_m)}{\sigma_N \sigma_{N_m}}$$

Assume $\sigma_N = 1$, $cov(N, N_m) = 1$

$$r_{NN_m} = \frac{1}{\sigma_{N_m}}$$

$$r_{DD_m} = \frac{cov(D, D_m)}{\sigma_D \sigma_{D_m}}$$

Assume $\sigma_D = 1$, $cov(D, D_m) = 1$

$$r_{DD_m} = \frac{1}{\sigma_{D_m}}$$

Given that we've assumed an additive noise model, we assume that the covariance between measured depressive symptoms and measured neural measures of reward processing is the same as the covariance between the reliability-corrected values. With these simplifications in place the relationship between measured correlation and observed correlation is greatly simplified.

$$r_{ND} = \frac{cov(ND)}{\sigma_N \sigma_D}$$

Since $\sigma_N = 1$ and $\sigma_D = 1$

$$r_{ND} = cov(ND)$$

$$r_{N_m D_m} = \frac{cov(N_m D_m)}{\sigma_{N_m} \sigma D_m}$$

Assume $cov(N_m D_m) = cov(ND) = r_{ND}$

$$r_{N_m D_m} = r_{ND} \cdot r_{NN_m} \cdot r_{DD_m}$$

$$r_{ND} = \frac{r_{N_m D_m}}{r_{NN_m} \cdot r_{DD_m}}$$

While these assumptions are necessary to make this estimation tractable, they do represent an optimistic scenario. If we define a set of past reliabilities ($r_{NN_m past}$ and $r_{DD_m past}$)and a set of expected future reliabilities in a planned study ($r_{NN_m future}$ and $r_{DD_m future}$), we can express the relationship between past observed correlation ($r_{N_m D_m past}$) and future expected correlation ($r_{N_m D_m future}$) .

$$r_{N_m D_m future} = \frac{r_{N_m D_m past}}{r_{NN_m past} \cdot r_{DD_m past}} \cdot r_{NN_m future} \cdot r_{DD_m future}$$

With these equations in hand, we can use the delta rule (9) to combine the estimated standard errors across the three measured correlations and derive confidence intervals for our estimate of $r_{N_m D_m future}$. Specifically:

$$\sigma_{r_{N_m D_m future}} = \sqrt{r_{N_m D_m future} \left(\frac{\sigma_{r_{NN_m past}}}{r_{NN_m past}}\right)^2 \left(\frac{\sigma_{r_{DD_m past}}}{r_{DD_m past}}\right)^2}$$

And the confidence interval is derived from the standard error with a Bonferroni correction for the number of estimated values, giving a critical z-score of 2.39. When estimating $r_{N_m D_m}$ for some future study, if we assume that the reliability of depression measures remains the same, then the r_{DD_m} terms cancel out and we only need to correct for 2 comparisons, instead of 3, giving a critical z-score of 2.24.

We calculated the relationship between past test-retest fMRI test-reliability for 1000 values of $r_{NN_m past}$ between 0 and 1 and 6 values of $r_{NN_m future}$ between 0.4 and 0.9. We did these calculations once with the assumption that the test-retest reliability of depression measures would be the same in past and future studies (Figure 4 of the main text). We also ran these calculations assuming that the test-retest reliability of depression measures would be 0.9 in a future study (Figure S8). In addition to estimating expected observed effect size, we also estimated sample size required to have 80% power with an alpha of 0.05 for a two-sided test that the Pearson's *r* is different from 0 (10).

<u>Review of previous meta-analyses</u>

We included three meta-analyses in our review of prior evidence for cross-sectional associations between reward processing and depression, Ng et al. (11), Keren, O'Callaghan et al. (1), and Zhang et al. (12). We did not include results from the comprehensive review of cognitive and emotional tasks conducted by Müller et al. (13) even though it did include reward processing tasks because the reward processing tasks were not analyzed separately.

Results of Zhang *et al.* were reproduced based on published coordinates and volumes. We focused on the that they report in figure 1 and table 3 of their publication, which they refer to as "Results from the global ALE analyses of reward-related processing in MDD". Results of Keren, O'Callaghan *et al.* were made available by the author. We focused on the results they report in figure 1A which are the results of whole brain studies reporting activity during reward feedback. Results of Ng *et al.* were downloaded from NeuroVault (14). We focus on the results they report in Figure 2A, which depicts results from 22 studies reporting less activity in response to reward in people with major depressive disorder (MDD) than healthy control and Figure 2B, which depicts results from 18 studies reporting greater activity in people with MDD. Results of Keren, O'Callaghan *et al.*, and Zhang *et al.* were transformed from Tailairach to FSL's MNI_152_T1 space via non-linear warp calculated and applied with AFNI (15). Resulting maps were visualized with MRIcroGL (16).

Supplemental Tables

Table S1: Set of reward processing studies included across Zhang et al. (17)**, Keren, O'Callaghan et al.** (1)**, and Ng et al.** (11)**.** 1 indicates a study was included in that review, 0 indicates it was not included. [1] indicates that the study was among those included in the analyses considered in Figure 1 A and B.

**Study**	**Zhang et al.**	**Keren, O'Callaghan et al.**	**Ng et al.**
Admon et al. (18)	0	1[1]	0
Arrondo et al. (19)	0	1	1[1]
Bremner et al. (20)	0	0	1[1]
Burger et al. (21)	0	0	1
Canli et al. (22)	1[1]	0	0
Casement et al. (23)	0	1	0
Chan et al. (24)	0	1	0
Chandrasekhar Pammi et al. (25)	0	1	0
Chantiluke et al. (26)	1[1]	0	1[1]
Chase et al. (27)	0	0	1[1]
Chung & Barch (28)	0	1	0
Demenescu et al. (29)	0	0	1[1]
Derntl et al. (30)	1[1]	0	0
Dichter et al. (31)	1[1]	1[1]	1[1]
Dillon et al. (32)	0	1[1]	0
Elliott et al. (33)	0	0	1[1]
Engelmann et al. (34)	0	0	1
Epstein et al. (35)	1[1]	0	0
Felder et al. (36)	0	1	0
Forbes et al. (37)	1[1]	1	0
Forbes et al. (38)	0	1	0
Fournier et al. (2013)	1[1]	0	1[1]
Fu et al. (39)	0	0	1[1]
Fu et al. (40)	1[1]	0	1[1]
Fu et al. (41)	0	0	1[1]
Gorka et al. (42)	0	1	0
Gotlib et al. (43)	1[1]	0	1[1]

Study	Zhang et al.	Keren, O'Callaghan et al.	Ng et al.
Gotlib et al. (44)	0	1	0
Gradin et al. (45)	1[1]	1[1]	0
Gradin et al. (46)	0	0	1[1]
Hagele et al. (47)	0	1	0
Hall et al. (48)	0	0	1[1]
Johnston et al. (49)	0	1[1]	1[1]
Keedwell et al. (50)	1[1]	0	1[1]
Knutson et al. (51)	1[1]	1[1]	1[1]
Kumar et al. (52)	1[1]	0	0
Kumari et al. (53)	1[1]	0	1[1]
Laurent et al. (54)	0	0	1
Liu et al. (55)	0	0	1
Luking et al. (56)	0	1[1]	0
McCabe et al. (57)	1[1]	0	0
Mitterschiffthaler et al. (58)	1[1]	0	0
Mori et al. (59)	0	1	0
Murrough et al. (60)	0	0	1
Olino et al. (61)	0	1	0
Olino et al. (62)	0	1	0
Pizzagalli et al. (63)	1[1]	1[1]	1[1]
Redlich et al. (64)	0	1	0
Remijnse et al. (65)	1[1]	1[1]	1[1]
Rizvi et al. (66)	0	0	1[1]
Robinson et al. (67)	1[1]	1[1]	0
Rosenblau et al. (68)	0	0	1
Rzepa et al. (69)	0	1	0
Satterthwaite et al. (70)	0	1	0
Scheuerecker et al. (71)	0	0	1
Schiller et al. (72)	0	1	1
Segarra et al. (73)	0	1[1]	1[1]

Study	Zhang et al.	Keren, O'Callaghan et al.	Ng et al.
Sharp et al. (74)	0	1[1]	1[1]
Smoski et al. (75)	1[1]	1[1]	1[1]
Smoski et al. (76)	1[1]	1[1]	1[1]
Steele et al. (77)	0	1	0
Stoy et al. (78)	0	1	0
Stringaris et al. (79)	0	1	0
Surguladze et al. (80)	1[1]	0	1[1]
Surguladze et al. (81)	0	0	1[1]
Townsend et al. (82)	0	0	1
Ubl et al. (83)	0	1	0
Ubl et al. (84)	0	1	0
Wagner et al. (85)	0	0	1[1]
Wang et al. (86)	0	0	1
Yang et al. (87)	0	1	0
Young et al. (88)	0	0	1[1]
Zhang et al. (89)	0	0	1[1]
Zhong et al. (90)	0	0	1

Table S2: Demographic information from longitudinal studies.
BA: Behavioral Analysis; CBT: Cognitive Behavioral Therapy; MDD: Major Depressive Disorder

Study	Hypothesis	Population	Age (Years)	Female	N	Mean Interval (Days)	Treatment
Admon et al. (18)	fMRI Global Treat	MDD	Adult	50.00%[1]	14	84	SAMe (5), escitalopram (5), placebo (4)
Bakker et al. (91)	fMRI Global Treat	Low-moderate risk	20.9 (2.1)	82.76%	87	7.5	reward anticipation on activity pleasantness
Bakker et al. (91)	fMRI Global Obs.	Low-moderate risk	20.9 (2.1)	82.76%	87	7.5	reward anticipation on activity pleasantness
Barch et al. (92)	EEG Global Treat	MDD	5.5 (0.8)	35.00%	60	126	Parent-Child Interaction Therapy
Barch et al. (92)	EEG RewP Treat	MDD	5.5 (0.8)	35.00%	60	126	Parent-Child Interaction Therapy
Bertocci et al. (93)	fMRI Global Obs.	Parent with BD or Axis-1	14.0 (2.3)	46.34%	41	29.6	
Bress et al. (94)	EEG RewP Obs., EEG Global Obs.	HV enriched for parental MDD	16	100.00%	61	630	
Burani et al. (95)	EEG RewP Treat, EEG Global Treat	Community	12.6 (1.7)	100.00%	183	365.25	sleep and stress
Burani et al. (95)	EEG RewP Obs., EEG Global Obs.	Community	12.6 (1.7)	100.00%	183	365.25	sleep and stress
Burkhouse et al. (96) CBT	EEG RewP Treat, EEG Global Treat	R-DOC internalizing symptoms	28.7 (8.9)	73.50%	34	84	CBT
Burkhouse et al. (96) Sertraline	EEG RewP Treat, EEG Global Treat	R-DOC internalizing symptoms	24.9 (8.1)	75.90%	29	84	sertraline
Flores et al. (97)	fMRI Global Obs.	HV	16.3 (1.5)	65.00%	34	7	
Greenberg et al. (98)	fMRI Global Treat	MDD	36.9 (12.8)	66.95%	194	56	sertraline or placebo
Greenberg et al. (98)	fMRI Global Obs.	MDD	36.9 (12.8)	66.95%	200	56	sertraline or placebo
Hasler et al. (99)	fMRI Striatum Obs., fMRI Global Obs.	Community	20	0.00%	93	730.5	
Jin et al. (100) High-risk	fMRI Global Obs.	High-risk	15.24 (0.58)[1]	100.00%	49	270	
Jin et al. (100) High-risk	fMRI Striatum Obs.	High-risk	15.24 (0.58)[1]	100.00%	49	270	
Jin et al. (100) Low-risk	fMRI Global Obs.	Low-risk	15.24 (0.58)[1]	100.00%	180	270	
Jin et al. (100) Low-risk	fMRI Striatum Obs.	Low-risk	15.24 (0.58)[1]	100.00%	180	270	
Kujawa et al. (101)	EEG RewP Treat, EEG Global Treat	Community	9	43.90%	369	1095.75	maternal depression

Study	Hypothesis	Population	Age (Years)	Female	N	Mean Interval (Days)	Treatment
Kujawa et al. (101)	EEG RewP Obs., EEG Global Obs.	Community	9	43.90%	369	1095.75	maternal depression
Kujawa et al. (102)	EEG RewP Treat, EEG Global Treat	Anxiety disorder + comorbidity	13.1 (4.0)	40.70%	22	84	CBT or sertraline
Langenecker et al. (103)	fMRI Global Treat	MDD	28.1 (9.9)	50.00%	10	84	duloxetine
Langenecker et al. (103)	fMRI Striatum Treat	MDD	28.1 (9.9)	50.00%	10	84	duloxetine
Luo et al. (104)	EEG RewP Obs.	HV	18.9 (0.2)	48.00%	25	180	
Luo et al. (104)	EEG Global Obs.	HV	18.9 (0.2)	48.00%	23	180	
Mackin et al. (105)	EEG RewP Obs., EEG Global Obs.	HV	14.4 (0.6)	100.00%	467	540	
Morgan et al. (106) Early Puberty	fMRI Striatum Obs., fMRI Global Obs.	Early puberty	11-13[1]	55.56%[1]	23	730.5	
Morgan et al. (106) Late Puberty	fMRI Striatum Obs., fMRI Global Obs.	Late puberty	11-13[1]	55.56%[1]	38	730.5	
Queirazza et al. (107)	fMRI Global Treat	MDD	39.2 (12.9)	48.65%	26	90	computerized CBT
Queirazza et al. (107)	fMRI Striatum Treat	MDD	39.2 (12.9)	48.65%	26	90	computerized CBT
Scult, et al. (108)	fMRI Striatum Obs., fMRI Global Obs.	Community excl. psychotic	19.9	68.00%	91	210	
Stringaris et al. (79)	fMRI Striatum Obs., fMRI Global Obs.	Community	14.4	56.07%	915	730.5	
Swartz et al. (109)	fMRI Global Obs.	Spectrum of MDD risk	16.9 (0.6)	50.76%	262	365.25	
Swartz et al. (109)	fMRI Striatum Obs.	Spectrum of MDD risk	16.9 (0.6)	50.76%	262	365.25	
Telzer et al. (110)	fMRI Striatum Obs., fMRI Global Obs.	Community	16.1	58.97%	39	365.25	
Walsh et al. (111)	fMRI Global Treat	MDD	33.0 (7.1)	71.05%	186	52.50[2]	BA

[1]indicates statistics reported for the entire study population, not for the subgroup upon which displayed prediction is based.

[2]slope of biweekly assessments over course of 15 weeks assessed with mixed effects model

Table S3: Prediction (i.e. longitudinal association) information for longitudinal studies.
OFC: Orbitofrontal Cortex; FRN: Feedback Related Negativity; RewP: Reward Positivity; dACC: dorsal Anterior Cingulate Cortex; MID: Monetary Incentive Delay task; NSUE: Non-Significant Unreported Effect

Study	Reward Type	Task	Contrast	ROI	Statistic	Value	r
Admon et al. (18)	monetary	MID	dACC-caudate connectivity during gain - dACC-caudate connectivity during loses	caudate	r	0.56	0.56
Bakker et al. (91)	monetary	reinforcement learning	reward prediction error	right putamen	b	0.05	0.33
Bakker et al. (91)	monetary	reinforcement learning	reward prediction error	right putamen	b	0.19	0.19
Barch et al. (92)	points	doors	RewP		t	2.09	0.26
Barch et al. (92)	points	doors	RewP		NSUE		
Bertocci et al. (93)	monetary	card guessing task	gain - neutral	mean beta from 15 significant clusters across the brain, no striatal clusters	NSUE		
Bress et al. (94)	monetary	doors with concurrent negative mood induction	FN		F	-5.46	-0.46
Burani et al. (95)	monetary	doors	RewP		B	0	-0.22
Burani et al. (95)	monetary	doors	RewP		B	-0.2	-0.07
Burkhouse et al. (96) CBT	monetary	guessing	RewP		t	-2.04	-0.34
Burkhouse et al. (96) Setraline	monetary	guessing	RewP		t	0.75	0.14
Flores et al. (97)	social	social reward	high positive - neutral	right posterior superior temporal sulcus/ temporoparietal junction	r	0.48	0.48
Greenberg et al. (98)	monetary	card guessing task	reward index	left ventral striatum	F	12.93	0.25
Greenberg et al. (98)	monetary	card guessing task	reward index	right orbitofrontal cortex	F	6.28	0.17
Hasler et al. (99)	monetary	card guessing task	gain - baseline	ventral striatum	r	-0.08	-0.08
Jin et al. (100) High-risk	monetary	doors	loss - baseline	OFC	r	-0.37	-0.37
Jin et al. (100) High-risk	monetary	doors	loss - baseline	striatum	NSUE		
Jin et al. (100) Low-risk	monetary	doors	loss - baseline	OFC	r	0.02	0.02

Study	Reward Type	Task	Contrast	ROI	Statistic	Value	r
Jin et al. (100) Low-risk	monetary	doors	loss - baseline	striatum	NSUE		
Kujawa et al. (101)	monetary	doors	RewP		b	-0.12	-0.1
Kujawa et al. (101)	monetary	doors	RewP		b	-0.07	-0.12
Kujawa et al. (102)	monetary	doors	RewP gains		t	-2.1	-0.42
Langenecker et al. (103)	monetary	MID	gain - neutral	right inferior frontal gyrus	z	-3.53	
Langenecker et al. (103)	monetary	MID	gain - neutral	putamen	z	-3.1	
Luo et al. (104)	monetary	MID with self and charitable outcomes	FRN		t	-1.27	-0.25
Luo et al. (104)	monetary	MID with self and charitable outcomes	eudaimonic anticipation vs neutral		F	5.36	0.44
Mackin et al. (105)	monetary	doors	RewP		b	-0.1	-0.12
Morgan et al. (106) Early Puberty	monetary	card guessing task	reward anticipation - baseline		r	0	0
Morgan et al. (106) Late Puberty	monetary	card guessing task	reward anticipation - baseline	caudate	t	-3.23	-0.47
Queirazza et al. (107)	points	Probabilistic reversal-learning task	parametric weighed RPE	cluster in right amygdala and right hippocampus	r	-0.64	-0.64
Queirazza et al. (107)	points	Probabilistic reversal-learning task	parametric weighed RPE	cluster in right putamen and caudate	r	-0.56	-0.56
Scult, et al. (108)	monetary	card guessing task	positive feedback > negative feedback	bilateral ventral striatum	b	-0.09	-0.11
Stringaris et al. (79)	monetary	MID	anticipation of large win versus anticipation of no win	left ventral striatum	t	-2.28	-0.08
Swartz et al. (109)	monetary	MID	gain - neutral anticipation	mean of bilateral ventral striatum small volume corrected clusters with significant activation	B	4.17	0.16

Study	Reward Type	Task	Contrast	ROI	Statistic	Value	r
Swartz et al. (109)	monetary	MID	gain - neutral anticipation	mean of bilateral ventral striatum small volume corrected clusters with significant activation	B	-0.83	-0.06
Telzer et al. (110)	monetary	family donation task	Costly donation > control	ventral striatum	B	-5.3	-0.45
Walsh et al. (111)	monetary	MID	gain - neutral	right putamen	t	2.82	0.2

Table S4: Assessment of prediction quality

Study	Out of Sample	Comprehensive CV	Sample Size	N	Multiple fit metrics	Fit Metrics	R^2 method	CV Method
Scult, et al. (108)	2	NA	1	91	2	B only	NA	NA
Jin et al. (100) High-risk	1[1]	0	2	49	1	r	2	0
Jin et al. (100) Low-risk	1[1]	0	1	180	1	r	2	0
Flores et al. (97)	2	NA	2	34	1	r	2	NA
Burkhouse et al. (96) CBT	2	NA	2	34	2	B, t	NA	NA
Burkhouse et al. (96) Setraline	2	NA	2	29	2	B, t	NA	NA
Bakker et al. (91)	2	NA	1	87	2	β	NA	NA
Luo et al. (104)	2	NA	2	25	1	B, t, R^2	2	NA
Luo et al. (104)	2	NA	2	25	2	B, t	NA	NA
Barch et al. (92)	2	NA	1	60	2	B, t	NA	NA
Barch et al. (92)	2	NA	2	44	1	B, t	NA	NA
Kujawa et al. (101)	2	NA	0	369	2	β	NA	NA
Langenecker et al. (103)	2	NA	2	10	2	Voxel Z	NA	NA
Bertocci et al. (93)	1[2]	1[5]	1	55	0	β, Sum of squared error	NA	1[6]
Swartz et al. (109)	2	NA	0	262	2	B	NA	NA
Goldstein et al. (112)	2	NA	0	369	2	B, t	NA	NA
Burani et al. (95)	2[3]	NA	1	183	2	B	NA	NA
Mackin et al. (105)	2	NA	0	467	2	B	NA	NA
Kujawa et al. (102)	2	NA	2	27	2	B, t	NA	NA
Walsh et al. (111)	2	NA	2	38	1	t, pseudo R^2	2	NA
Queirazza et al. (107)	2[4]	NA	2	37	1	r	2	NA
Greenberg et al. (98)	2	NA	0	222	2	F	NA	NA
Admon et al. (18)	2	NA	2	14	1	ΔF, ΔR^2	2	NA
Stringaris et al. (79)	2	NA	0	915	2	β	NA	NA
Bress et al. (94)	2	NA	1	68	2	F, β	NA	NA
Morgan et al. (106) Early Puberty	2	NA	2	23	1	t, r	2	NA
Morgan et al. (106) Late Puberty	2	NA	2	40	1	r	2	NA
Telzer et al. (110)	2	NA	2	39	2	B, β	NA	NA
Hasler et al. (99)	2	NA	1	93	1	r	NA	NA

[1]Cross validation used for orbital loss model
[2]Non-significant unreported effect size, coefficient was pushed to 0 by elastic net, cross validation was used
[3]Used a bootstrap approach to generate confidence intervals, but did not use it for out of sample testing. DMN rated as 2 and G'OC rated as 1, reconciled to 2
[4]Used a CV method for treatment response as a binary, but not for severity
[5]Cross validated, but unclear which steps
[6]Cross validation scheme not specified

Table S5: Assessment of adherence to open science practices

Study	Preregistration	Preregistration repository	Preregistration ID	Shared Code	Shared Data
Scult, et al. (108)	2			2	2
Jin et al. (100) High-risk	2			2	2
Jin et al. (100) Low-risk	2			2	2
Flores et al. (97)	2			2	2
Burkhouse et al. (96) CBT	1	ClinicalTrials.gov	NCT01903447	2	2
Burkhouse et al. (96) Setraline	1	ClinicalTrials.gov	NCT01903447	2	2
Bakker et al. (91)	1	trialregister.nl	3662	2	2
Luo et al. (104)	2			2	2
Luo et al. (104)	2			2	2
Barch et al. (92)	1	ClinicalTrials.gov	NCT02076425	2	2
Barch et al. (92)	1	ClinicalTrials.gov	NCT02076425	2	2
Kujawa et al. (101)	2			2	2
Langenecker et al. (103)	2			2	2
Bertocci et al. (93)	2			2	2
Swartz et al. (109)	2			2	2
Goldstein et al. (112)	2			2	2
Burani et al. (95)	2			2	2
Mackin et al. (105)	2			2	2
Kujawa et al. (102)	2			2	2
Walsh et al. (111)	2			2	2
Queirazza et al. (107)	2			2	2
Greenberg et al. (98)	1	PubMed	PMC6100771, PMC5485858	2	2
Admon et al. (18)	2			2	2
Stringaris et al. (79)	2			2[1]	2[1]
Bress et al. (94)	2			2	2
Morgan et al. (106) Early Puberty	2			2	2
Morgan et al. (106) Late Puberty	2			2	2
Telzer et al. (110)	2			2	2
Hasler et al. (99)	2			2	2

[1]DMN and GO'C disagreed about these ratings since information on data access (https://imagen-europe.com/resources/imagen-dataset/) and code (https://github.com/imagen2) is available online, but was not referenced in the publication.

Table S6: Summary of predictive meta-analytic hypotheses of treatment effects.

Modality	Specificity	Design	k	r (95% CI)	z	p	i^2	Worst r	Worst z	Worst p
fMRI	Striatum	Treat	2							
EEG	RewP	Treat	6	-0.16 [-0.27, -0.05]	-2.85	0.0044	25.62%	-0.13	-1.89	0.059
fMRI	Global	Treat	6	0.36 [0.18, 0.52]	4.30		54.53%	0.28	2.86	
EEG	Global	Treat	6	0.20 [0.10, 0.29]	3.88		24.75%	0.18	2.93	

The "global" results are best-case analyses taking the absolute value of strongest effect from any reward-related analysis to define the upper bounds of the relationship between reward processing and future changes in depression. p-values are not given because significant difference from 0 is trivial after taking the absolute value. The least significant results from a leave-one-out analysis are shown in the "worst" columns. No meta-analysis was done on striatal fMRI predicting treatment outcomes because only two studies were found.

Table S7: Test-retest reliability of fMRI measures of neural reward processing based on information collated in Elliott et al. (8).

Study	Task	Interval (days)	n	Thresholded	Reliability
Chase et al., (113)	Reward	7	37	No	0.284
Fliessbach et al., (114)	Reward (adapted MID)	8	25	No	0.136
Fliessbach et al., (114)	Reward (box guessing)	8	25	No	0.286
Fliessbach et al., (114)	Reward (number guessing)	8	25	No	0.29
Holiga et al., (115)	MID	14	30	No	0.58
Plitcha et al., (116)	Monetary reward anticipation	15	25	No	0.591
Schlagenhauf, (117)	MID	28	10	No	0.502
Keren et al., (118)	MID	80	18	Yes	0.801
Elliott et al., (8)	MID	79	20	No	0.45

Table S8: Test-retest reliability of clinical symptom measures found by informal review. Interval is the interval between assessments in days.

Study	Assessment	Population	r	n	Interval (days)
Langvik, E. et al. (119)	SHAPS	psychology students	0.71	94	70
Watson, D (120)	IDAS-II-Dysphoria	college students	0.74	841	14
Sprinkle et al. (121)	BDI-II	undergraduates with initial appointment at a clinic	0.96	46	3.2
Harvey, P.D et al. (122)	ALS-anxiety-depression	undergraduate female students	0.57	28	28
Harvey, P.D et al. (122)	ALS-anxiety-depression	undergraduate male students	0.81	26	28
Gerson et al. (123)	CALS	inpatients of child and adolescent psychiatric...	0.68	35	14
Gerson et al. (123)	CALS	students suburban school	0.89	72	14
CF Saylor et al (124)	CDI	school students 5th-6th graders	0.38	69	7
CF Saylor et al (124)	CDI	children with emotional problems	0.87	30	7
CF Saylor et a.l (124)	CDI	children with emotional problems	0.59	24	42
Smucker, M.R et al. (125)	CDI	female elementary school students	0.74	78	21
Smucker, M.R et al. (125)	CDI	male elementary school students	0.77	77	21
Achenbach TM (126)	YSR(6-18)-internalizing subscale	non referred children	0.8	89	8

Supplemental Figures

Figure S1: Schematic of reward processing and the Monetary Incentive Delay (MID) Task.

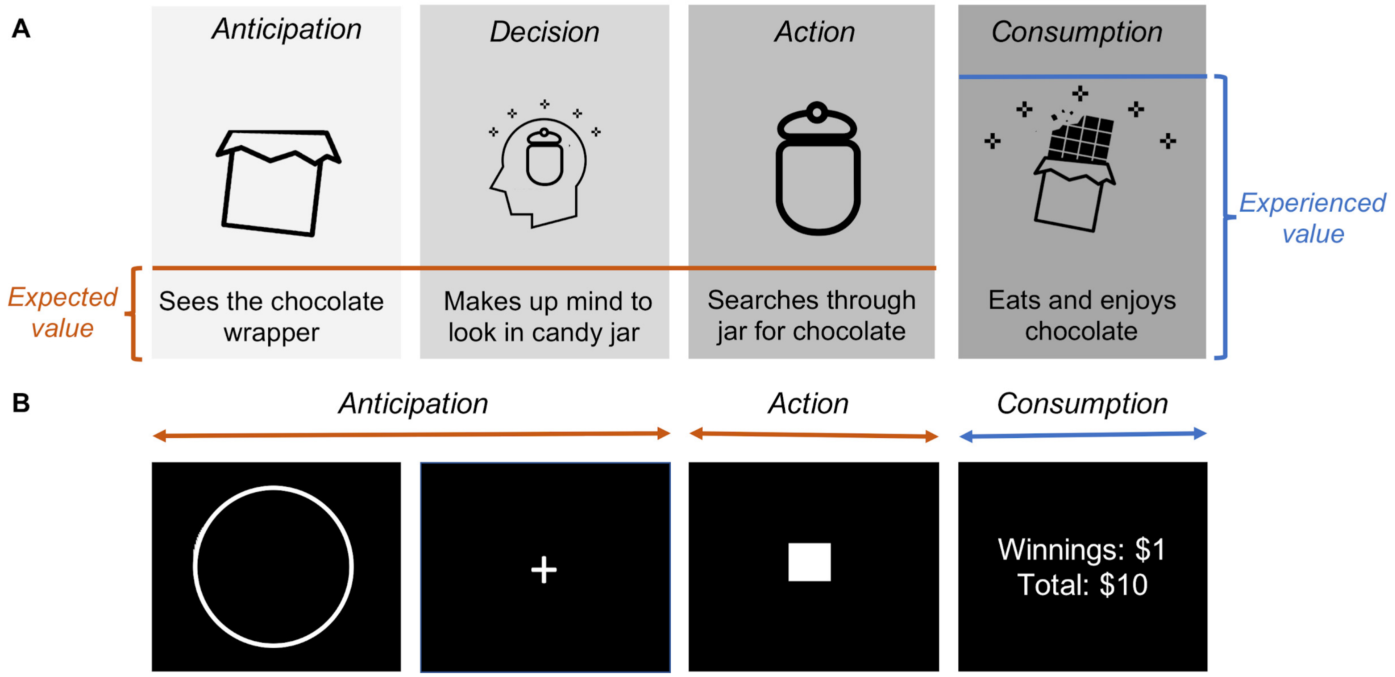

(A) depicts an everyday example of reward processing. A child sees a chocolate wrapper on her kitchen table and forms the *expectation* that there is chocolate nearby. In other words, she forms a *prediction* and *anticipates* that she will find chocolate. The orange line represents her expectation of the reward. The child *decides* to investigate a nearby candy jar on the kitchen table. She then tries to open it based on her prediction that it may contain chocolate. Here, the child *acts* and expends *effort* to obtain a reward. The child finds one chocolate left and receives hedonic pleasure, which constitutes the consummatory component of reward processing. The child experiences what is termed a positive *reward prediction error (RPE)*, because the experienced value of the chocolate is greater than the expected value. RPEs are thought to underlie reward-related learning, which for this example, would increase the likelihood that the child looks into the candy jar for chocolate in the future. **(B)** depicts the structure of the MID task, the most commonly used task for accessing reward processing. In this task participants are first presented one of three symbols indicating that the trial is a loss, gain, or neutral trial, in this case a gain trial is illustrated. They are then shown a fixation cross, and after a variable interval, they are presented with a target. The subject must respond within a certain amount of time in order to win the reward on a gain trial or avoid losing money on a loss trial. The last component of the trial is feedback, in which they are shown the result of the trial.

Figure S2: Forest plot for random effects meta-analysis of observational EEG studies reporting a reward positivity (RewP) effect for the correlation with change in depressive symptoms. We found that the mean effect size was -0.17 [-0.30, -0.04]. The size of the marker corresponds to the number of participants in the study. The error bars indicate the 95% confidence interval of the estimated correlation based on the sample size.

Study	Population	Age (years)	% Female	n
Bress et al. (94)	HV enriched for parental MDD	16.0	100.00%	68
Mackin et al. (105)	HV	14.4 (0.6)	100.00%	467
Luo et al. (104)	HV	18.9 (0.2)	48.00%	25
Kujawa et al. (101)	Community	9.0	43.90%	369
Burani et al. (95)	Community	12.6 (1.7)	100.00%	183
EEG RewP Obs. ($I^2 = 74.42$, $p = 0.011$)				

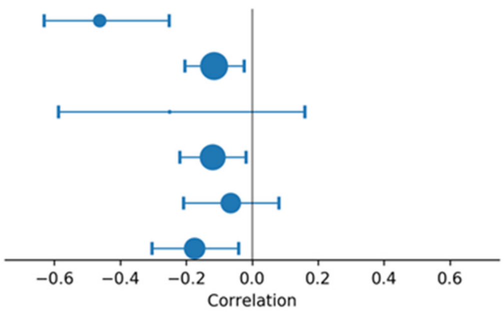

Figure S3: Forest plot for random effects meta-analysis of observational fMRI studies reporting any effect for the correlation with change in depressive symptoms. We found that the mean effect size was 0.17 [0.09, 0.24]. The size of the marker corresponds to the number of participants in the study. The error bars indicate the 95% confidence interval of the estimated correlation based on the sample size. The results in lighter blue represent null effects where the effect-size was imputed via MetaNSUE. Since we were comparing across activation, psychophysiological interactions, and changes in connectivity, we took the absolute value of the reported effects. p-values should be disregarded because significant difference from 0 is trivial after taking the absolute value. [1] indicates statistics reported for the entire study population, not for the subgroup upon which displayed prediction is based.

Study	Population	Age (years)	% Female	n
Morgan et al. (106)	Early puberty	11-13[1]	55.56%[1]	23
Morgan et al. (106)	Late puberty	11-13[1]	55.56%[1]	40
Telzer et al. (110)	Community	16.1	58.97%	39
Stringaris et al. (79)	Community	14.4	56.07%	915
Jin et al. (100)	High-risk	15.24 (0.58)[1]	100.00%	49
Jin et al. (100)	Low-risk	15.24 (0.58)[1]	100.00%	180
Hasler et al. (99)	Community	20.0	0.00%	93
Flores et al. (97)	HV	16.3 (1.5)	65.00%	34
Greenberg et al. (98)	MDD	36.9 (12.8)	66.95%	222
Bertocci et al. (93)	Parent with BD or Axis-1	14.0 (2.3)	46.34%	41
Scult, et al. (108)	Community excl. psychotic	19.9	68.00%	91
Bakker et al. (91)	Low-moderate risk	20.9 (2.1)	82.76%	87
Swartz et al. (109)	Spectrum of MDD risk	16.9 (0.6)	50.76%	262
fMRI Global Obs. ($I^2 = 51.18$, $p = 1.5e-05$)				

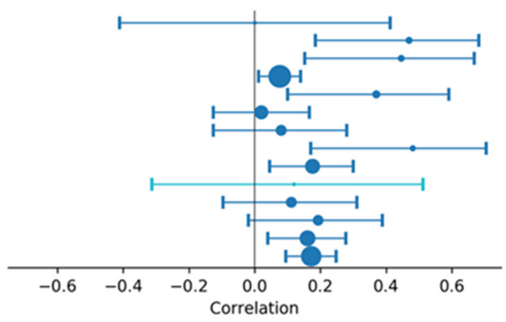

Figure S4: Forest plot for random effects meta-analysis of observational EEG studies any effect for the correlation with change in depressive symptoms. We found that the mean effect size was 0.20 [0.04, 0.34]. Since we were comparing across multiple signals and analyses, we took the absolute value of the reported effects. p-values should be disregarded because significant difference from 0 is trivial after taking the absolute value. The error bars indicate the 95% confidence interval of the estimated correlation based on the sample size.

Study	Population	Age (years)	% Female	n
Bress et al. (94)	HV enriched for parental MDD	16.0	100.00%	68
Mackin et al. (105)	HV	14.4 (0.6)	100.00%	467
Luo et al. (104)	HV	18.9 (0.2)	48.00%	25
Kujawa et al. (101)	Community	9.0	43.90%	369
Burani et al. (95)	Community	12.6 (1.7)	100.00%	183
EEG Global Obs. ($I^2 = 81.46$, $p = 0.011$)				

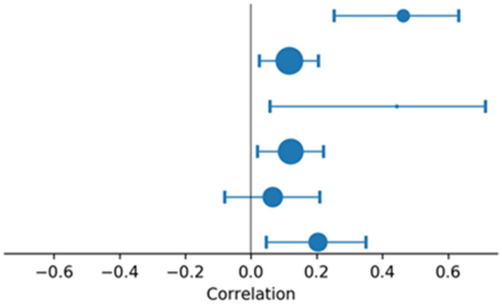

Figure S5: Forest plot for random effects meta-analysis of treatment studies with EEG reporting a reward positivity (RewP) effect for the correlation with change in depressive symptoms. We found that the mean effect size was -0.16 [-0.27, -0.05]. The size of the marker corresponds to the number of participants in the study. The error bars indicate the 95% confidence interval of the estimated correlation based on the sample size. The results in lighter blue represent null effects where the effect-size was imputed via MetaNSUE.

Study	Population	Treatment	Age (years)	% Female	n
Burkhouse et al. (96)	R-DOC internalizing symptoms	CBT	28.7 (8.9)	73.50%	34
Burkhouse et al. (96)	R-DOC internalizing symptoms	sertraline	24.9 (8.1)	75.90%	29
Kujawa et al. (101)	Community	maternal depression	9.0	43.90%	369
Kujawa et al. (102)	Anxiety disorder + comorbidity	CBT or sertraline	13.1 (4.0)	40.70%	27
Burani et al. (95)	Community	sleep and stress	12.6 (1.7)	100.00%	183
Barch et al. (92)	MDD	Parent-Child Interaction Therapy	5.5 (0.8)	35.00%	60
EEG RewP Treat ($I^2 = 26.14$, $p = 0.0049$)					

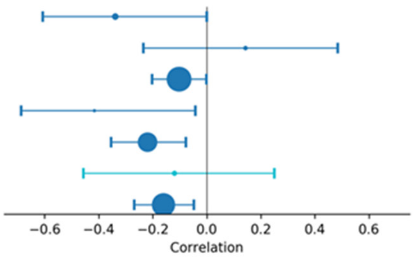

Figure S6: Forest plot for random effects meta-analysis of treatment studies with fMRI reporting any effect for the correlation with change in depressive symptoms. We found that the mean effect size was 0.36 [0.18, 0.52]. Since we were comparing across activation, psychophysiological interactions, and changes in connectivity, we took the absolute value of the reported effects. p-values should be disregarded because significant difference from 0 is trivial after taking the absolute value. The size of the marker corresponds to the number of participants in the study. The error bars indicate the 95% confidence interval of the estimated correlation based on the sample size. The results in lighter blue represent null effects where the effect-size was imputed via MetaNSUE.

Study	Population	Treatment	Age (years)	% Female	n
Admon et al. (18)	MDD	SAMe (5), escitalopram (5), placebo (4)	Adult	50.00%[1]	14
Walsh et al. (111)	MDD	BA	33.0 (7.1)	71.05%	38
Greenberg et al. (98)	MDD	sertraline or placebo	36.9 (12.8)	66.95%	222
Queirazza et al. (107)	MDD	computerized CBT	39.2 (12.9)	48.65%	37
Bakker et al. (91)	Low-moderate risk	reward anticipation on activity pleasantness	20.9 (2.1)	82.76%	87
Langenecker et al. (103)	MDD	duloxetine	28.1 (9.9)	50.00%	10
fMRI Global Treat ($I^2 = 54.59$, $p = 0.00012$)					

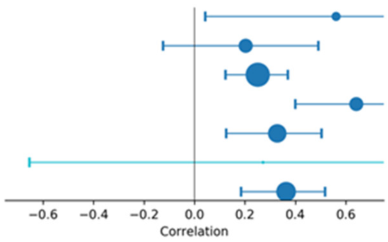

Figure S7: Forest plot for random effects meta-analysis of treatment studies with EEG reporting any effect for the correlation with change in depressive symptoms. We found that the mean effect size was 0.20 [0.10, 0.29]. Since we were comparing across activation, psychophysiological interactions, and changes in connectivity, we took the absolute value of the reported effects. p-values should be disregarded because significant difference from 0 is trivial after taking the absolute value.

Study	Population	Treatment	Age (years)	% Female	n
Burkhouse et al. (96)	R-DOC internalizing symptoms	CBT	28.7 (8.9)	73.50%	34
Burkhouse et al. (96)	R-DOC internalizing symptoms	sertraline	24.9 (8.1)	75.90%	29
Kujawa et al. (101)	Community	maternal depression	9.0	43.90%	369
Kujawa et al. (102)	Anxiety disorder + comorbidity	CBT or sertraline	13.1 (4.0)	40.70%	27
Burani et al. (95)	Community	sleep and stress	12.6 (1.7)	100.00%	183
Barch et al. (92)	MDD	Parent-Child Interaction Therapy	5.5 (0.8)	35.00%	60
EEG Global Treat ($I^2 = 24.75$, $p = 0.00011$)					

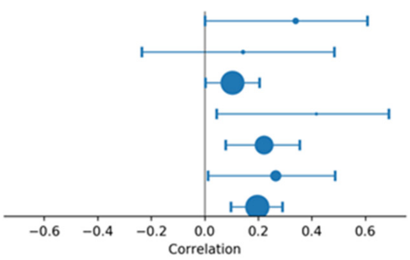

Figure S8: Impact of fMRI test-retest reliability on expected effect size in planned studies assuming a higher test-retest reliability of depression measures. in our meta-analysis of observational studies of the association between striatal fMRI signal and change in depression symptoms, we found a correlation of -0.10 [-0.18, -0.03] (Figure 2, Table 1). If we planned a new study, our estimate of the observed effect size would depend on our belief about the reliability of the studies used to estimate the effect, and the test-retest reliability of both fMRI and depression measures in our planned study. This figure depicts the correlation of change in depression and reward processing we would expect to observe given different estimates for the test-retest reliability of the studies in our meta-analysis at different levels of reliability in the planned study. We estimated the test-retest reliability of fMRI reward processing tasks to be 0.44 [0.28, 0.57] from nine studies (Table S7). The minimum and maximum values in these studies are the limits of x-axis. For this figure, we assume a depressive symptom measurement reliability of 0.9 instead of the 0.77 [0.67,0.84] based on eight studies (Table S8) that we depict in Figure 4. The most optimistic case (**dotted line**) is that these previous studies were at the lower end of this range of reliability with a test-retest reliability of 0.28, and that our planned study will have a reliability of 0.8. In this situation we would expect to observe a correlation between reward processing and change in depressive symptoms of -0.33 [-0.61, 0.01] and required sample size of 67 to have 80% power to detect with a two-sided test for Pearson correlation difference from 0. A second case (**solid line**) is that previous studies had a reliability of 0.44 and our planned study will increase this 0.7, resulting in an observed correlation of -0.19 [-0.37, 0.01] and requiring a sample size of 217. Finally, a pessimistic case (**dashed line**) is that previous studies had a reliability of 0.57 and that our planned study will have a reliability of 0.6, resulting in an observed correlation of -0.12 [-0.24, -0.003] and requiring a sample size of 507.

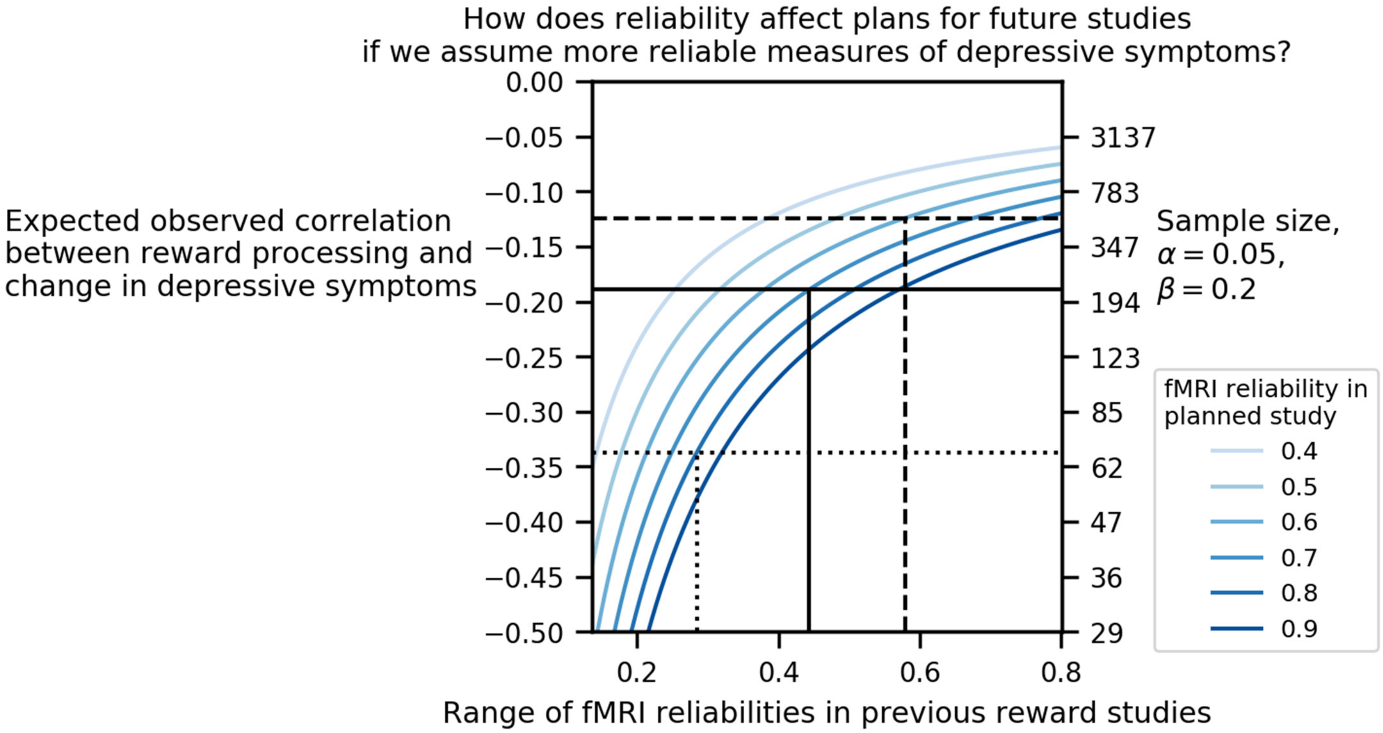

Figure S9: Diversity of tasks and contrasts in studies reviewed by Ng *et al.* (11)**, Keren, O'Callaghan *et al.*** (1)**, and Zhang *et al.*** (9)**.** The majority of task/contrast combinations in the literature have appeared in only a single study (54 out of 69). Classification of the task as assessing anticipation or feedback is based on the information reported in each meta-analysis. Tasks are sorted by the frequency of report. Contrasts within tasks are also sorted by the frequency of report. MID: Monetary Incentive Delay task; EEfRT: Effort-Expenditure for Rewards Task; RPE: Reward Prediction Error

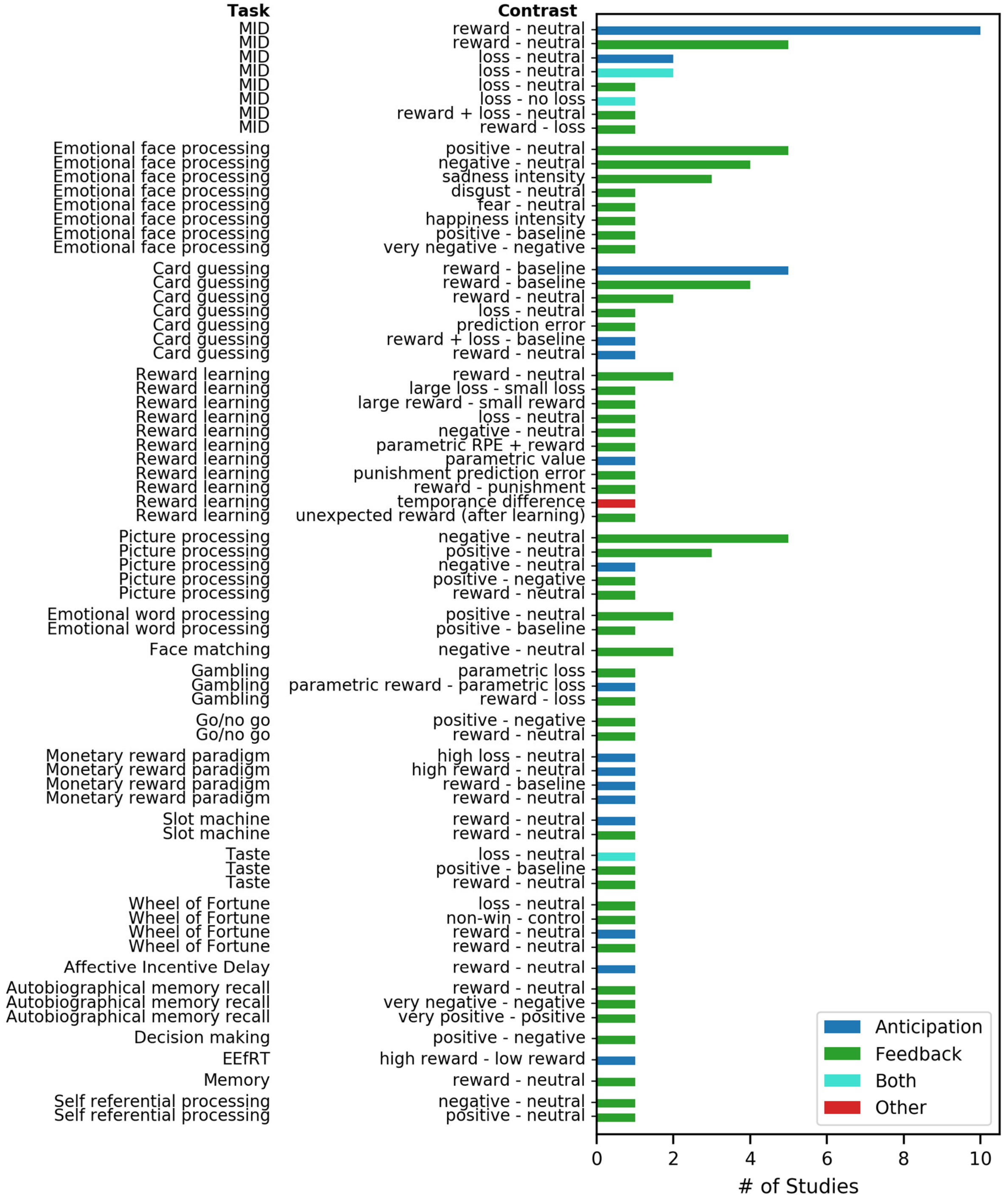

Figure S10: PRISMA diagram for review of longitudinal studies of reward processing and depression.

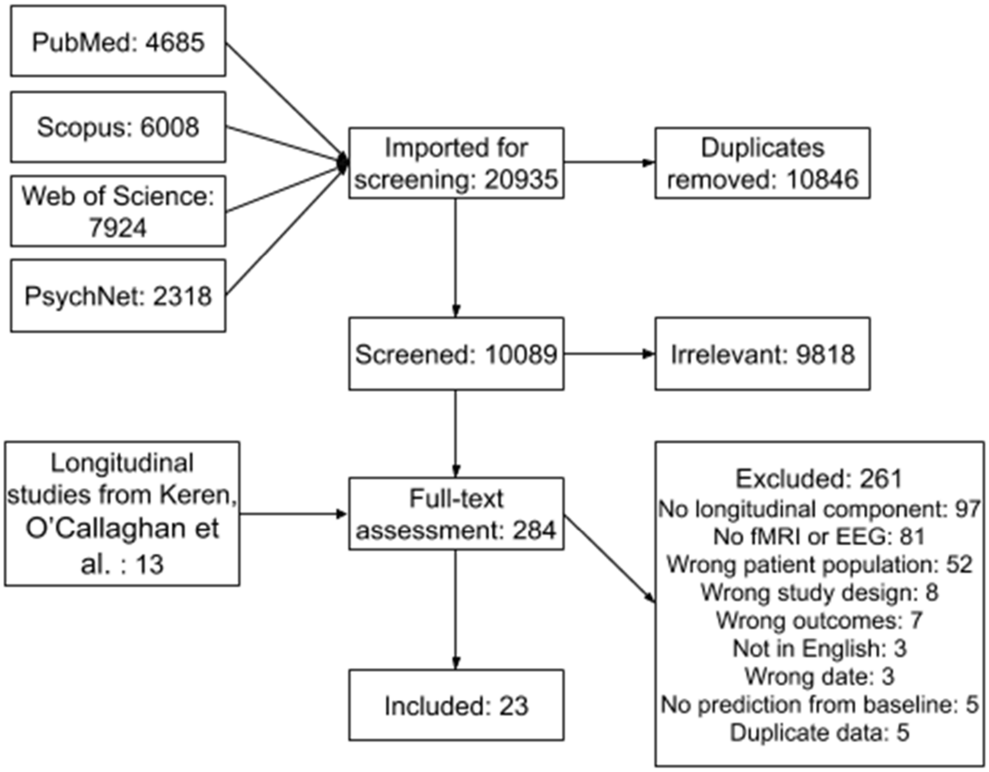

Supplemental Boxes

Box S1: Contextualizing effect sizes

Throughout the paper we use three typically-employed metrics to help understand the magnitude and direction of the relationship between reward processing abnormalities (RPAs) and depression. As has recently been pointed out (127), even effect sizes that are typically considered small (e.g. correlation r = 0.3), could have profound implications for behavior. We therefore contextualize effect sizes by providing some examples from outside psychology below,

Pearson correlation *r*: We apply correlation correlation to quantify the relationship between RPAs and future depressive symptoms in **Longitudinal Association**. It represents a summary of the strength of the linear relationship between two variables. The magnitude is between 0 (no relationship) and 1 (perfect correlation) and the sign indicates the direction of the relationship.
Cohen's *d*: We apply this to the *EEG* portion of **Cross-Sectional Association** to quantify the difference in EEG responses to reward between depressed and healthy participants. The metric represents the mean difference between groups divided by the pooled standard deviation. A Cohen's *d* of 1 corresponds to a difference of 1 standard deviation between groups.
Area under the receiver operating characteristic curve (AUC): We use this in the **Cross-Sectional Association** section to assess how well reward processing distinguishes depressed from non-depressed participants. The receiver operating characteristic curve is the plot of true positive rate versus false positive rate across the range of possible values of the independent variable. AUC may be between 0 and 1. An AUC of 0.5 corresponds to chance performance.

Effect sizes of familiar associations:

When assessing the magnitude of an effect size, it is helpful to consider some examples (7,127,128):

- Effect of ibuprofen on pain reduction: r = 0.14, d = 0.28, AUC = 0.58
- Gender and weight for US adults: r = 0.26, d = 0.54, AUC = 0.65
- Weight and height for US adults: r = 0.44, d = 0.98, AUC = 0.76
- Gender and height for US adults: r = 0.67, d = 1.81, AUC = 0.90

Box S2: Explanation versus prediction

The two goals of this review highlight two different philosophical approaches to psychiatric, and medical research more generally: one concerned with discovering the causal chain of events, the other with predicting outcomes. We expand on these approaches below.

Explanation: The first of the two approaches, in this context, seeks to answer whether reward processing abnormalities (RPAs) are a cause of depression. Many different models (see Figure 4) may fit a given set of observations better than the null model of no relationship. Therefore the strongest support of an *a priori* explanatory model will come from a demonstration that it is superior to both the null and to reasonable alternatives (129,130).

Prediction: The other main approach seeks to determine if RPAs could be a clinical marker of future depression severity. This is a predictive question and does not depend on a mechanistic understanding of depression. Prediction is used here in a particular sense, namely as an to answer the question "How well could I predict depression severity in a new group of subjects (an out-of-sample test)?"

Incremental Validity: When testing if RPAs are useful for predicting depression severity, it is important to compare the predictive ability against a meaningful alternative, such as the baseline clinical scores. Demonstrating the incremental validity of a prediction demonstrates that the added predictors contribute useful additional information (129,131).

Finally, it is important to note that explanatory and predictive analyses should inform each other. If RPAs were shown to be strongly predictive of changes in depression, then future explanatory studies should be designed to understand the structure of that relationship.

Supplemental References

1. Keren H, O'Callaghan G, Vidal-Ribas P, Buzzell GA, Brotman MA, Leibenluft E, *et al.* (2018): Reward Processing in Depression: A Conceptual and Meta-Analytic Review Across fMRI and EEG Studies. *Am J Psychiatry* 175: 1111–1120.

2. Richards JM, Plate RC, Ernst M (2013): A systematic review of fMRI reward paradigms used in studies of adolescents vs. adults: The impact of task design and implications for understanding neurodevelopment. *Neurosci Biobehav Rev* 37: 976–991.

3. Poldrack RA, Huckins G, Varoquaux G (2019): Establishment of Best Practices for Evidence for Prediction: A Review. *JAMA Psychiatry*. https://doi.org/10.1001/jamapsychiatry.2019.3671

4. Friedman H (1982): Simplified determinations of statistical power, magnitude of effect and research sample sizes. *Educ Psychol Meas* 42: 521–526.

5. Albajes-Eizagirre A, Solanes A, Radua J (2018): Meta-analysis of non-statistically significant unreported effects: *Stat Methods Med Res*. https://doi.org/10.1177/0962280218811349

6. Radua J, Schmidt A, Borgwardt S, Heinz A, Schlagenhauf F, McGuire P, Fusar-Poli P (2015): Ventral Striatal Activation During Reward Processing in Psychosis: A Neurofunctional Meta-Analysis. *JAMA Psychiatry* 72: 1243–1251.

7. Salgado JF (2018): Transforming the Area under the Normal Curve (AUC) into Cohen's d, Pearson's r pb , Odds-Ratio, and Natural Log Odds-Ratio: Two Conversion Tables. *Eur J Psychol Appl Leg Context* 10: 35–47.

8. Elliott ML, Knodt AR, Ireland D, Morris ML, Poulton R, Ramrakha S, *et al.* (2020): What is the test-retest reliability of common task-fMRI measures? New empirical evidence and a meta-analysis. *bioRxiv* 681700.

9. Casella G, Berger RL (2002): *Statistical Inference*. Duxbury Thomson Learning. Retrieved from https://books.google.com/books?id=ZpkPPwAACAAJ

10. Lachin JM (1981): Introduction to sample size determination and power analysis for clinical trials. *Control Clin Trials* 2: 93–113.

11. Ng TH, Alloy LB, Smith DV (2019): Meta-analysis of reward processing in major depressive disorder reveals distinct abnormalities within the reward circuit. *Transl Psychiatry* 9: 1–10.

12. Zhang W-N, Chang S-H, Guo L-Y, Zhang K-L, Wang J (2013): The neural correlates of reward-related processing in major depressive disorder: A meta-analysis of functional magnetic resonance imaging studies. *J Affect Disord* 151: 531–539.

13. Müller VI, Cieslik EC, Serbanescu I, Laird AR, Fox PT, Eickhoff SB (2017): Altered Brain Activity in Unipolar Depression Revisited: Meta-analyses of Neuroimaging Studies. *JAMA Psychiatry* 74: 47–55.

14. Gorgolewski KJ, Varoquaux G, Rivera G, Schwarz Y, Ghosh SS, Maumet C, *et al.* (2015): NeuroVault.org: a web-based repository for collecting and sharing unthresholded statistical maps of the human brain. *Front Neuroinformatics* 9. https://doi.org/10.3389/fninf.2015.00008

15. Cox RW (1996): AFNI: Software for Analysis and Visualization of Functional Magnetic Resonance Neuroimages. *Comput Biomed Res* 29: 162–173.

16. Rorden C, Karnath H-O, Bonilha L (2007): Improving Lesion-Symptom Mapping. *J Cogn Neurosci* 19: 1081–1088.

17. Zhang W-N, Chang S-H, Guo L-Y, Zhang K-L, Wang J (2013): The neural correlates of reward-related processing in major depressive disorder: A meta-analysis of functional magnetic resonance imaging studies. *J Affect Disord* 151: 531–539.

18. Admon R, Nickerson LD, Dillon DG, Holmes AJ, Bogdan R, Kumar P, *et al.* (2015): Dissociable cortico-striatal connectivity abnormalities in major depression in response to monetary gains and penalties. *Psychol Med* 45: 121–131.

19. Arrondo G, Segarra N, Metastasio A, Ziauddeen H, Spencer J, Reinders NR, *et al.* (2015): Reduction in ventral striatal activity when anticipating a reward in depression and schizophrenia: a replicated cross-diagnostic finding. *Front Psychol* 6. https://doi.org/10.3389/fpsyg.2015.01280

20. Bremner JD, Vythilingam M, Vermetten E, Charney DS (2007): Effects of antidepressant treatment on neural correlates of emotional and neutral declarative verbal memory in depression. *J Affect Disord* 101: 99–111.

21. Bürger C, Redlich R, Grotegerd D, Meinert S, Dohm K, Schneider I, *et al.* (2017): Differential Abnormal Pattern of Anterior Cingulate Gyrus Activation in Unipolar and Bipolar Depression: an fMRI and Pattern Classification Approach. *Neuropsychopharmacology* 42: 1399–1408.

22. Canli T, Sivers H, Thomason ME, Whitfield-Gabrieli S, Gabrieli JDE, Gotlib IH (2004): Brain activation to emotional words in depressed vs healthy subjects. *NeuroReport* 15: 2585–2588.

23. Casement MD, Keenan KE, Hipwell AE, Guyer AE, Forbes EE (2016): Neural Reward Processing Mediates the Relationship between Insomnia Symptoms and Depression in Adolescence. *Sleep* 39: 439–447.

24. Chan RCK, Li Z, Li K, Zeng Y, Xie W, Yan C, *et al.* (2016): Distinct processing of social and monetary rewards in late adolescents with trait anhedonia. *Neuropsychology* 30: 274–280.

25. Chandrasekhar Pammi VS, Pillai Geethabhavan Rajesh P, Kesavadas C, Rappai Mary P, Seema S, Radhakrishnan A, Sitaram R (2015): Neural loss aversion differences between depression patients and healthy individuals: A functional MRI investigation. *Neuroradiol J* 28: 97–105.

26. Chantiluke K, Halari R, Simic M, Pariante CM, Papadopoulos A, Giampietro V, Rubia K (2012): Fronto-Striato-Cerebellar Dysregulation in Adolescents with Depression During Motivated Attention. *Biol Psychiatry* 71: 59–67.

27. Chase HW, Nusslock R, Almeida JR, Forbes EE, LaBarbara EJ, Phillips ML (2013): Dissociable patterns of abnormal frontal cortical activation during anticipation of an uncertain reward or loss in bipolar versus major depression. *Bipolar Disord* 15: 839–854.

28. Chung YS, Barch D (2015): Anhedonia is associated with reduced incentive cue related activation in the basal ganglia. *Cogn Affect Behav Neurosci* 15: 749–767.

29. Demenescu LR, Renken R, Kortekaas R, Tol M-J van, Marsman JBC, Buchem MA van, *et al.* (2011): Neural correlates of perception of emotional facial expressions in out-patients with mild-to-moderate depression and anxiety. A multicenter fMRI study. *Psychol Med* 41: 2253–2264.

30. Derntl B, Seidel E-M, Eickhoff SB, Kellermann T, Gur RC, Schneider F, Habel U (2011): Neural correlates of social approach and withdrawal in patients with major depression. *Soc Neurosci* 6: 482–501.

31. Dichter GS, Kozink RV, McClernon FJ, Smoski MJ (2012): Remitted major depression is characterized by reward network hyperactivation during reward anticipation and hypoactivation during reward outcomes. *J Affect Disord* 136: 1126–1134.

32. Dillon DG, Dobbins IG, Pizzagalli DA (2014): Weak reward source memory in depression reflects blunted activation of VTA/SN and parahippocampus. *Soc Cogn Affect Neurosci* 9: 1576–1583.

33. Elliott R, Rubinsztein JS, Sahakian BJ, Dolan RJ (2002): The Neural Basis of Mood-Congruent Processing Biases in Depression. *Arch Gen Psychiatry* 59: 597–604.

34. Engelmann JB, Berns GS, Dunlop BW (2017): Hyper-responsivity to losses in the anterior insula during economic choice scales with depression severity. *Psychol Med* 47: 2879–2891.

35. Epstein J, Pan H, Kocsis JH, Yang Y, Butler T, Chusid J, *et al.* (2006): Lack of Ventral Striatal Response to Positive Stimuli in Depressed Versus Normal Subjects. *Am J Psychiatry* 163: 1784–1790.

36. Felder JN, Smoski MJ, Kozink RV, Froeliger B, McClernon J, Bizzell J, *et al.* (2012): Neural mechanisms of subclinical depressive symptoms in women: a pilot functional brain imaging study. *BMC Psychiatry* 12: 152.

37. Forbes EE, Hariri AR, Martin SL, Silk JS, Moyles DL, Fisher PM, *et al.* (2009): Altered Striatal Activation Predicting Real-World Positive Affect in Adolescent Major Depressive Disorder. *Am J Psychiatry* 166: 64–73.

38. Forbes EE, Ryan ND, Phillips ML, Manuck SB, Worthman CM, Moyles DL, *et al.* (2010): Healthy Adolescents' Neural Response to Reward: Associations With Puberty, Positive Affect, and Depressive Symptoms. *J Am Acad Child Adolesc Psychiatry* 49: 162-172.e5.

39. Fu CHY, Williams SCR, Cleare AJ, Brammer MJ, Walsh ND, Kim J, *et al.* (2004): Attenuation of the Neural Response to Sad Faces in Major Depressionby Antidepressant Treatment: A Prospective, Event-Related Functional Magnetic Resonance ImagingStudy. *Arch Gen Psychiatry* 61: 877–889.

40. Fu CHY, Williams SCR, Brammer MJ, Suckling J, Kim J, Cleare AJ, *et al.* (2007): Neural Responses to Happy Facial Expressions in Major Depression Following Antidepressant Treatment. *Am J Psychiatry* 164: 599–607.

41. Fu CHY, Williams SCR, Cleare AJ, Scott J, Mitterschiffthaler MT, Walsh ND, *et al.* (2008): Neural Responses to Sad Facial Expressions in Major Depression Following Cognitive Behavioral Therapy. *Biol Psychiatry* 64: 505–512.

42. Gorka SM, Huggins AA, Fitzgerald DA, Nelson BD, Phan KL, Shankman SA (2014): Neural response to reward anticipation in those with depression with and without panic disorder. *J Affect Disord* 164: 50–56.

43. Gotlib IH, Sivers H, Gabrieli JDE, Whitfield-Gabrieli S, Goldin P, Minor KL, Canli T (2005): Subgenual anterior cingulate activation to valenced emotional stimuli in major depression. *NeuroReport* 16: 1731–1734.

44. Gotlib IH, Hamilton JP, Cooney RE, Singh MK, Henry ML, Joormann J (2010): Neural Processing of

Reward and Loss in Girls at Risk for Major Depression. *Arch Gen Psychiatry* 67: 380–387.

45. Gradin VB, Kumar P, Waiter G, Ahearn T, Stickle C, Milders M, *et al.* (2011): Expected value and prediction error abnormalities in depression and schizophrenia. *Brain* 134: 1751–1764.

46. Gradin VB, Pérez A, MacFarlane JA, Cavin I, Waiter G, Engelmann J, *et al.* (2015): Abnormal brain responses to social fairness in depression: an fMRI study using the Ultimatum Game. *Psychol Med* 45: 1241–1251.

47. Hägele C, Schlagenhauf F, Rapp M, Sterzer P, Beck A, Bermpohl F, *et al.* (2015): Dimensional psychiatry: reward dysfunction and depressive mood across psychiatric disorders. *Psychopharmacology (Berl)* 232: 331–341.

48. Hall GBC, Milne AMB, MacQueen GM (2014): An fMRI study of reward circuitry in patients with minimal or extensive history of major depression. *Eur Arch Psychiatry Clin Neurosci* 264: 187–198.

49. Johnston BA, Tolomeo S, Gradin V, Christmas D, Matthews K, Douglas Steele J (2015): Failure of hippocampal deactivation during loss events in treatment-resistant depression. *Brain* 138: 2766–2776.

50. Keedwell PA, Andrew C, Williams SCR, Brammer MJ, Phillips ML (2005): A Double Dissociation of Ventromedial Prefrontal Cortical Responses to Sad and Happy Stimuli in Depressed and Healthy Individuals. *Biol Psychiatry* 58: 495–503.

51. Knutson B, Bhanji JP, Cooney RE, Atlas LY, Gotlib IH (2008): Neural responses to monetary incentives in major depression. *Biol Psychiatry* 63: 686–692.

52. Kumar P, Waiter G, Ahearn T, Milders M, Reid I, Steele JD (2008): Abnormal temporal difference reward-learning signals in major depression. *Brain* 131: 2084–2093.

53. Kumari V, Mitterschiffthaler MT, Teasdale JD, Malhi GS, Brown RG, Giampietro V, *et al.* (2003): Neural abnormalities during cognitive generation of affect in Treatment-Resistant depression. *Biol Psychiatry* 54: 777–791.

54. Laurent HK, Ablow JC (2013): A Face a Mother Could Love: Depression-Related Maternal Neural Responses to Infant Emotion Faces. *Soc Neurosci* 8: 228–239.

55. Liu W-H, Valton V, Wang L-Z, Zhu Y-H, Roiser JP (2017): Association between habenula dysfunction and motivational symptoms in unmedicated major depressive disorder. *Soc Cogn Affect Neurosci* 12: 1520–1533.

56. Luking KR, Pagliaccio D, Luby JL, Barch DM (2016): Depression Risk Predicts Blunted Neural Responses to Gains and Enhanced Responses to Losses in Healthy Children. *J Am Acad Child Adolesc Psychiatry* 55: 328–337.

57. McCabe C, Cowen PJ, Harmer CJ (2009): Neural representation of reward in recovered depressed patients. *Psychopharmacology (Berl)* 205: 667–677.

58. Mitterschiffthaler MT, Kumari V, Malhi GS, Brown RG, Giampietro VP, Brammer MJ, *et al.* (2003): Neural response to pleasant stimuli in anhedonia: an fMRI study. *NeuroReport* 14: 177–182.

59. Mori A, Okamoto Y, Okada G, Takagaki K, Jinnin R, Takamura M, *et al.* (2016): Behavioral activation can normalize neural hypoactivation in subthreshold depression during a monetary incentive delay task. *J Affect Disord* 189: 254–262.

60. Murrough JW, Collins KA, Fields J, DeWilde KE, Phillips ML, Mathew SJ, *et al.* (2015): Regulation of neural responses to emotion perception by ketamine in individuals with treatment-resistant major depressive disorder [no. 2]. *Transl Psychiatry* 5: e509–e509.

61. Olino TM, McMakin DL, Dahl RE, Ryan ND, Silk JS, Birmaher B, *et al.* (2011): "I won, but I'm not getting my hopes up": Depression moderates the relationship of outcomes and reward anticipation. *Psychiatry Res Neuroimaging* 194: 393–395.

62. Olino TM, McMakin DL, Morgan JK, Silk JS, Birmaher B, Axelson DA, *et al.* (2014): Reduced reward anticipation in youth at high-risk for unipolar depression: A preliminary study. *Dev Cogn Neurosci* 8: 55–64.

63. Pizzagalli DA, Holmes AJ, Dillon DG, Goetz EL, Birk JL, Bogdan R, *et al.* (2009): Reduced Caudate and Nucleus Accumbens Response to Rewards in Unmedicated Individuals With Major Depressive Disorder. *Am J Psychiatry* 166: 702–710.

64. Redlich R, Dohm K, Grotegerd D, Opel N, Zwitserlood P, Heindel W, *et al.* (2015): Reward Processing in Unipolar and Bipolar Depression: A Functional MRI Study. *Neuropsychopharmacology* 40: 2623–2631.

65. Remijnse PL, Nielen MMA, Balkom AJLM van, Hendriks G-J, Hoogendijk WJ, Uylings HBM, Veltman DJ (2009): Differential frontal–striatal and paralimbic activity during reversal learning in major depressive disorder and obsessive–compulsive disorder. *Psychol Med* 39: 1503–1518.

66. Rizvi SJ, Salomons TV, Konarski JZ, Downar J, Giacobbe P, McIntyre RS, Kennedy SH (2013): Neural response to emotional stimuli associated with successful antidepressant treatment and behavioral activation. *J Affect Disord* 151: 573–581.

67. Robinson OJ, Cools R, Carlisi CO, Sahakian BJ, Drevets WC (2012): Ventral Striatum Response During Reward and Punishment Reversal Learning in Unmedicated Major Depressive Disorder. *Am J Psychiatry* 169: 152–159.

68. Rosenblau G, Sterzer P, Stoy M, Park S, Friedel E, Heinz A, *et al.* (2012): Functional neuroanatomy of emotion processing in major depressive disorder is altered after successful antidepressant therapy. *J Psychopharmacol (Oxf)* 26: 1424–1433.

69. Rzepa E, Fisk J, McCabe C (2017): Blunted neural response to anticipation, effort and consummation of reward and aversion in adolescents with depression symptomatology. *J Psychopharmacol (Oxf)* 31: 303–311.

70. Satterthwaite TD, Kable JW, Vandekar L, Katchmar N, Bassett DS, Baldassano CF, *et al.* (2015): Common and Dissociable Dysfunction of the Reward System in Bipolar and Unipolar Depression. *Neuropsychopharmacology* 40: 2258–2268.

71. Scheuerecker J (2010): Orbitofrontal volume reductions during emotion recognition in patients with major depression. *J Psychiatry Neurosci* 35: 311–320.

72. Schiller CE, Minkel J, Smoski MJ, Dichter GS (2013): Remitted major depression is characterized by reduced prefrontal cortex reactivity to reward loss. *J Affect Disord* 151: 756–762.

73. Segarra N, Metastasio A, Ziauddeen H, Spencer J, Reinders NR, Dudas RB, *et al.* (2016): Abnormal Frontostriatal Activity During Unexpected Reward Receipt in Depression and Schizophrenia:

Relationship to Anhedonia. *Neuropsychopharmacology* 41: 2001–2010.

74. Sharp C, Kim S, Herman L, Pane H, Reuter T, Strathearn L (2014): Major depression in mothers predicts reduced ventral striatum activation in adolescent female offspring with and without depression. *J Abnorm Psychol* 123: 298–309.

75. Smoski MJ, Felder J, Bizzell J, Green SR, Ernst M, Lynch TR, Dichter GS (2009): fMRI of alterations in reward selection, anticipation, and feedback in major depressive disorder. *J Affect Disord* 118: 69–78.

76. Smoski MJ, Rittenberg A, Dichter GS (2011): Major depressive disorder is characterized by greater reward network activation to monetary than pleasant image rewards. *Psychiatry Res Neuroimaging* 194: 263–270.

77. Steele JD, Kumar P, Ebmeier KP (2007): Blunted response to feedback information in depressive illness. *Brain* 130: 2367–2374.

78. Stoy M, Schlagenhauf F, Sterzer P, Bermpohl F, Hägele C, Suchotzki K, *et al.* (2012): Hyporeactivity of ventral striatum towards incentive stimuli in unmedicated depressed patients normalizes after treatment with escitalopram. *J Psychopharmacol (Oxf)* 26: 677–688.

79. Stringaris A, Vidal-Ribas Belil P, Artiges E, Lemaitre H, Gollier-Briant F, Wolke S, *et al.* (2015): The Brain's Response to Reward Anticipation and Depression in Adolescence: Dimensionality, Specificity, and Longitudinal Predictions in a Community-Based Sample. *Am J Psychiatry* 172: 1215–1223.

80. Surguladze S, Brammer MJ, Keedwell P, Giampietro V, Young AW, Travis MJ, *et al.* (2005): A differential pattern of neural response toward sad versus happy facial expressions in major depressive disorder. *Biol Psychiatry* 57: 201–209.

81. Surguladze SA, El-Hage W, Dalgleish T, Radua J, Gohier B, Phillips ML (2010): Depression is associated with increased sensitivity to signals of disgust: A functional magnetic resonance imaging study. *J Psychiatr Res* 44: 894–902.

82. Townsend JD, Eberhart NK, Bookheimer SY, Eisenberger NI, Foland-Ross LC, Cook IA, *et al.* (2010): fMRI activation in the amygdala and the orbitofrontal cortex in unmedicated subjects with major depressive disorder. *Psychiatry Res Neuroimaging* 183: 209–217.

83. Ubl B, Kuehner C, Kirsch P, Ruttorf M, Flor H, Diener C (2015): Neural reward processing in individuals remitted from major depression. *Psychol Med* 45: 3549–3558.

84. Ubl B, Kuehner C, Kirsch P, Ruttorf M, Diener C, Flor H (2015): Altered neural reward and loss processing and prediction error signalling in depression. *Soc Cogn Affect Neurosci* 10: 1102–1112.

85. Wagner G, Schachtzabel C, Peikert G, Bär K-J (2015): The neural basis of the abnormal self-referential processing and its impact on cognitive control in depressed patients. *Hum Brain Mapp* 36: 2781–2794.

86. Wang L, Krishnan KR, Steffens DC, Potter GG, Dolcos F, McCarthy G (2008): Depressive State- and Disease-Related Alterations in Neural Responses to Affective and Executive Challenges in Geriatric Depression. *Am J Psychiatry* 165: 863–871.

87. Yang X, Huang J, Lan Y, Zhu C, Liu X, Wang Y, *et al.* (2016): Diminished caudate and superior temporal gyrus responses to effort-based decision making in patients with first-episode major depressive disorder. *Prog Neuropsychopharmacol Biol Psychiatry* 64: 52–59.

88. Young KD, Bodurka J, Drevets WC (2016): Differential neural correlates of autobiographical memory recall in bipolar and unipolar depression. *Bipolar Disord* 18: 571–582.

89. Zhang B, Li S, Zhuo C, Li M, Safron A, Genz A, *et al.* (2017): Altered task-specific deactivation in the default mode network depends on valence in patients with major depressive disorder. *J Affect Disord* 207: 377–383.

90. Zhong M, Wang X, Xiao J, Yi J, Zhu X, Liao J, *et al.* (2011): Amygdala hyperactivation and prefrontal hypoactivation in subjects with cognitive vulnerability to depression. *Biol Psychol* 88: 233–242.

91. Bakker JM, Goossens L, Kumar P, Lange IMJ, Michielse S, Schruers K, *et al.* (2019): From laboratory to life: associating brain reward processing with real-life motivated behaviour and symptoms of depression in non-help-seeking young adults. *Psychol Med* 49: 2441–2451.

92. Barch DM, Whalen D, Gilbert K, Kelly D, Kappenman ES, Hajcak G, Luby JL (2019): Neural Indicators of Anhedonia: Predictors and Mechanisms of Treatment Change in a Randomized Clinical Trial in Early Childhood Depression. *Biol Psychiatry* 85: 863–871.

93. Bertocci MA, Hanford L, Manelis A, Iyengar S, Youngstrom EA, Gill MK, *et al.* (2019): Clinical, cortical thickness and neural activity predictors of future affective lability in youth at risk for bipolar disorder: initial discovery and independent sample replication. *Mol Psychiatry* 24: 1856–1867.

94. Bress JN, Foti D, Kotov R, Klein DN, Hajcak G (2013): Blunted neural response to rewards prospectively predicts depression in adolescent girls. *Psychophysiology* 50: 74–81.

95. Burani K, Klawohn J, Levinson AR, Klein DN, Nelson BD, Hajcak G (2019): Neural Response to Rewards, Stress and Sleep Interact to Prospectively Predict Depressive Symptoms in Adolescent Girls. *J Clin Child Adolesc Psychol Off J Soc Clin Child Adolesc Psychol Am Psychol Assoc Div 53* 1–10.

96. Burkhouse KL, Gorka SM, Klumpp H, Kennedy AE, Karich S, Francis J, *et al.* (2018): Neural Responsiveness to Reward as an Index of Depressive Symptom Change Following Cognitive-Behavioral Therapy and SSRI Treatment. *J Clin Psychiatry* 79: 0–0.

97. Flores LE, Eckstrand KL, Silk JS, Allen NB, Ambrosia M, Healey KL, Forbes EE (2018): Adolescents' neural response to social reward and real-world emotional closeness and positive affect. *Cogn Affect Behav Neurosci* 18: 705–717.

98. Greenberg T, Fournier JC, Stiffler R, Chase HW, Almeida JR, Aslam H, *et al.* (2019): Reward related ventral striatal activity and differential response to sertraline versus placebo in depressed individuals. *Mol Psychiatry* 1–11.

99. Hasler BP, Casement MD, Sitnick SL, Shaw DS, Forbes EE (2017): Eveningness among late adolescent males predicts neural reactivity to reward and alcohol dependence 2 years later. *Behav Brain Res* 327: 112–120.

100. Jin J, Narayanan A, Perlman G, Luking K, DeLorenzo C, Hajcak G, *et al.* (2017): Orbitofrontal cortex activity and connectivity predict future depression symptoms in adolescence. *Biol Psychiatry Cogn Neurosci Neuroimaging* 2: 610–618.

101. Kujawa A, Hajcak G, Klein DN (2019): Reduced reward responsiveness moderates the effect of maternal depression on depressive symptoms in offspring: evidence across levels of analysis. *J Child Psychol Psychiatry* 60: 82–90.

102. Kujawa A, Burkhouse KL, Karich SR, Fitzgerald KD, Monk CS, Phan KL (2019): Reduced Reward Responsiveness Predicts Change in Depressive Symptoms in Anxious Children and Adolescents Following Treatment. *J Child Adolesc Psychopharmacol* 29: 378–385.

103. Langenecker SA, Klumpp H, Peters AT, Crane NA, DelDonno SR, Bessette KL, *et al.* (2019): Multidimensional imaging techniques for prediction of treatment response in major depressive disorder. *Prog Neuropsychopharmacol Biol Psychiatry* 91: 38–48.

104. Luo Y, Jiang H, Chen X, Zhang Y, You X (2019): Temporal dynamics of hedonic and eudaimonic reward processing: An event-related potentials (ERPs) study. *Int J Psychophysiol* 137: 63–71.

105. Mackin DM, Kotov R, Perlman G, Nelson BD, Goldstein BL, Hajcak G, Klein DN (20190502): Reward processing and future life stress: Stress generation pathway to depression. *J Abnorm Psychol* 128: 305.

106. Morgan JK, Olino TM, McMakin DL, Ryan ND, Forbes EE (2013): Neural response to reward as a predictor of increases in depressive symptoms in adolescence. *Neurobiol Dis* 52: 66–74.

107. Queirazza F, Fouragnan E, Steele JD, Cavanagh J, Philiastides MG (2019): Neural correlates of weighted reward prediction error during reinforcement learning classify response to cognitive behavioral therapy in depression. *Sci Adv* 5: eaav4962.

108. Scult MA, Knodt AR, Radtke SR, Brigidi BD, Hariri AR (2019): Prefrontal Executive Control Rescues Risk for Anxiety Associated with High Threat and Low Reward Brain Function. *Cereb Cortex* 29: 70–76.

109. Swartz JR, Weissman DG, Ferrer E, Beard SJ, Fassbender C, Robins RW, *et al.* (2020): Reward-Related Brain Activity Prospectively Predicts Increases in Alcohol Use in Adolescents. *J Am Acad Child Adolesc Psychiatry* 59: 391–400.

110. Telzer EH, Fuligni AJ, Lieberman MD, Galván A (2014): Neural sensitivity to eudaimonic and hedonic rewards differentially predict adolescent depressive symptoms over time. *Proc Natl Acad Sci* 111: 6600–6605.

111. Walsh E, Carl H, Eisenlohr-Moul T, Minkel J, Crowther A, Moore T, *et al.* (2017): Attenuation of Frontostriatal Connectivity During Reward Processing Predicts Response to Psychotherapy in Major Depressive Disorder. *Neuropsychopharmacology* 42: 831–843.

112. Goldstein BL, Kessel EM, Kujawa A, Finsaas MC, Davila J, Hajcak G, Klein DN (undefined/ed): Stressful life events moderate the effect of neural reward responsiveness in childhood on depressive symptoms in adolescence. *Psychol Med* 1–8.

113. Chase HW, Fournier JC, Greenberg T, Almeida JR, Stiffler R, Zevallos CR, *et al.* (2015): Accounting for Dynamic Fluctuations across Time when Examining fMRI Test-Retest Reliability: Analysis of a Reward Paradigm in the EMBARC Study. *PLOS ONE* 10: e0126326.

114. Fliessbach K, Rohe T, Linder NS, Trautner P, Elger CE, Weber B (2010): Retest reliability of reward-related BOLD signals. *NeuroImage* 50: 1168–1176.

115. Holiga Š, Sambataro F, Luzy C, Greig G, Sarkar N, Renken RJ, *et al.* (2018): Test-retest reliability of task-based and resting-state blood oxygen level dependence and cerebral blood flow measures. *PLOS ONE* 13: e0206583.

116. Plichta MM, Schwarz AJ, Grimm O, Morgen K, Mier D, Haddad L, *et al.* (2012): Test–retest reliability of evoked BOLD signals from a cognitive–emotive fMRI test battery. *NeuroImage* 60: 1746–1758.

117. Schlagenhauf F, Juckel G, Koslowski M, Kahnt T, Knutson B, Dembler T, *et al.* (2008): Reward system activation in schizophrenic patients switched from typical neuroleptics to olanzapine. *Psychopharmacology (Berl)* 196: 673–684.

118. Keren H, Chen G, Benson B, Ernst M, Leibenluft E, Fox NA, *et al.* (2018): Is the encoding of Reward Prediction Error reliable during development? *NeuroImage* 178: 266–276.

119. Langvik E, Borgen Austad S (2019): Psychometric Properties of the Snaith–Hamilton Pleasure Scale and a Facet-Level Analysis of the Relationship Between Anhedonia and Extraversion in a Nonclinical Sample. *Psychol Rep* 122: 360–375.

120. Watson D, Stanton K, Clark LA (2017): Self-report indicators of negative valence constructs within the research domain criteria (RDoC): A critical review. *J Affect Disord* 216: 58–69.

121. Sprinkle SD, Lurie D, Insko SL, Atkinson G, Jones GL, Logan AR, Bissada NN (2002): Criterion validity, severity cut scores, and test-retest reliability of the Beck Depression Inventory-II in a university counseling center sample. *J Couns Psychol* 49: 381.

122. Harvey PD, Greenberg BR, Serper MR (1989): The affective lability scales: Development, reliability, and validity. *J Clin Psychol* 45: 786–793.

123. Gerson AC, Gerring JP, Freund L, Joshi PT, Capozzoli J, Brady K, Denckla MB (1996): The Children's Affective Lability Scale: A psychometric evaluation of reliability. *Psychiatry Res* 65: 189–198.

124. Saylor CF, Finch AJ, Spirito A, Bennett B (19850101): The Children's Depression Inventory: A systematic evaluation of psychometric properties. *J Consult Clin Psychol* 52: 955.

125. Smucker MR, Craighead WE, Craighead LW, Green BJ (1986): Normative and reliability data for the children's depression inventory. *J Abnorm Child Psychol* 14: 25–39.

126. Achenbach TM, Rescorla LA (2001): *Manual for the ASEBA School-Age Forms and Profiles*. Burlington, VT: University of Vermont Research Center for Children, Youth, & Families.

127. Funder DC, Ozer DJ (2019): Evaluating Effect Size in Psychological Research: Sense and Nonsense. *Adv Methods Pract Psychol Sci* 2: 156–168.

128. Meyer GJ, Finn SE, Eyde LD, Kay GG, Moreland KL, Dies RR, *et al.* (2001): Psychological testing and psychological assessment: A review of evidence and issues. *Am Psychol* 56: 128–165.

129. Yarkoni T (2019): The Generalizability Crisis. https://doi.org/10.31234/osf.io/jqw35

130. Yarkoni T, Westfall J (2017): Choosing Prediction Over Explanation in Psychology: Lessons From Machine Learning. *Perspect Psychol Sci* 12: 1100–1122.

131. Shackman AJ, Fox AS (2018): Getting serious about variation: Lessons for clinical neuroscience. *Trends Cogn Sci* 22: 368–369.
